# PSMD9 promotes the malignant progression of hepatocellular carcinoma by interacting with c-Cbl to activate EGFR signaling and recycling

**DOI:** 10.1186/s13046-024-03062-3

**Published:** 2024-05-14

**Authors:** Yuting Su, Lili Meng, Chao Ge, Yuqi Liu, Chi Zhang, Yue Yang, Wei Tian, Hua Tian

**Affiliations:** 1 State Key Laboratory of Systems Medicine for Cancer, Shanghai Cancer Institute, Renji Hospital, Shanghai Jiao Tong University School of Medicine, 25/Ln 2200, Xietu Road, Shanghai, 200032 China; 2 Department of Pathology, Zhongshan Hospital, Fudan University, Shanghai, 200032 China; 3 Department of Pathology, The Affiliated Hospital of Youjiang Medical University for Nationalities, Baise, 533000 China; 4 The Key Laboratory of Molecular Pathology (Hepatobiliary Diseases) of Guangxi, Baise, 533000 China

**Keywords:** PSMD9, EGFR, c-Cbl, Progression, Hepatocellular carcinoma

## Abstract

**Background:**

Mounting evidences shows that the ubiquitin‒proteasome pathway plays a pivotal role in tumor progression. The expression of 26S proteasome non-ATPase regulatory subunit 9 (PSMD9) is correlated with recurrence and radiotherapy resistance in several tumor types. However, the role and mechanism of PSMD9 in hepatocellular carcinoma (HCC) progression remain largely unclear.

**Methods:**

PSMD9 was identified as a prognosis-related biomarker for HCC based on analysis of clinical characteristics and RNA-seq data from The Cancer Genome Atlas (TCGA), Gene Expression Omnibus (GEO) and the JP Project of the International Cancer Genome Consortium (ICGC-LIRI-JP). PSMD9 expression was analyzed in cancer tissues and adjacent noncancerous tissues via immunohistochemistry and Western blotting. Multiple in vivo and in vitro experimental techniques (such as CCK-8, colony formation, EdU, and Transwell assays; flow cytometry; Western blotting; quantitative RT-PCR; Coimmunoprecipitation assay and immunofluorescence confocal imaging) were used to assess the functions of PSMD9 in the pathogenesis of HCC.

**Results:**

We found that the expression of PSMD9 was upregulated and associated with a poor prognosis in HCC patients. PSMD9 promoted HCC cell proliferation, migration, invasion and metastasis. Knockdown of PSMD9 significantly inhibited HCC cell proliferation by inducing G1/S cell cycle arrest and apoptosis. Mechanistically, we demonstrated that PSMD9 promoted HCC cell proliferation and metastasis via direct interaction with the E3 ubiquitin ligase c-Cbl, suppresses EGFR ubiquitination, influenced EGFR endosomal trafficking and degradation and subsequently activated ERK1/2 and Akt signaling. In addition, we showed that PSMD9 knockdown sensitized HCC cells to the tyrosine kinase inhibitor erlotinib in vitro and in vivo.

**Conclusions:**

Collectively, our results indicate that PSMD9 drives HCC progression and erlotinib resistance by suppressing c-Cbl mediated EGFR ubiquitination and therefore can be a potential therapeutic target for HCC.

**Supplementary Information:**

The online version contains supplementary material available at 10.1186/s13046-024-03062-3.

## Introduction

Hepatocellular carcinoma (HCC) is the fourth most common cause of cancer-related death and ranks sixth in incidence among cancers worldwide. Surgical resection is recognized as an effective treatment for early-stage HCC [1]. Unfortunately, the prognosis of HCC remains poor because of its propensity for metastatic progression and poor response to pharmacological treatment. Therefore, there is an unmet need for the identification of novel diagnostic and therapeutic targets to improve the prognosis of HCC.

The epidermal growth factor receptor (EGFR) is one of four members of the EGFR/ErbB subfamily of receptor tyrosine kinases (RTKs). EGFR plays a key role in the development of various cancers [2]. Small molecule inhibitors targeting EGFR have been applied to treat solid tumors, such as lung cancer [3]. However, EGFR antibodies and inhibitors have not achieved satisfactory clinical results in HCC [4]. Thus, a better understanding of the EGFR signaling cascade in HCC is needed.

The ubiquitin‒proteasome (Ub) system plays an important role in oncogenesis, cancer development and chemoresistance [5]. The 26S proteasome is composed of a 20S core proteasome (CP) and a 19S regulatory particle (RP). It is the 19S regulator that links the 20S CP to the Ub system. The 19S RP consists of at least 18 different subunits and orchestrates all the steps that lead to the degradation of ubiquitylated proteins [6]. The proteasome degrades most cellular proteins in a tightly controlled manner, and thereby, its dysregulation is involved in multiple diseases, including cancer. The proteasome has become an attractive target for therapy in many cancers. Several proteasome inhibitors have exhibited marked antitumor effects [7, 8]. Our previous study showed that Rpn10/PSMD4 (a 19S regulator) promotes tumor progression by regulating hypoxia-inducible factor 1alpha through the PTEN/Akt signaling pathway in HCC [9]. Rpn10 may be a novel therapeutic target in HCC. PSMD9 (Rpn4) encodes a non-ATPase subunit of the 19S regulator. Many studies have shown that PSMD9 plays an important role in tumor progression. PSMD9 expression is correlated with recurrence after radiotherapy in patients with cervical cancer [10]. PSMD9 expression predicts the response to radiotherapy in breast cancer patients [11]. PSMD9 is associated with radiotherapy resistance and shorter survival in bone metastatic prostate cancer patients [12]. PSMD9 is implicated in ribosomal protein shuttling to the nucleolus and subsequent activation of p53 [13], which allows cells with PSMD9 to overcome the nucleolar stress induced by anticancer drugs and gain a survival advantage. However, the involvement of PSMD9 in HCC progression remains unknown. Thus, we explored the role of PSMD9 in HCC progression and the underlying molecular mechanism. In this study, we revealed that PSMD9 drives HCC progression and erlotinib resistance by decreasing c-Cbl-mediated EGFR ubiquitination.

## Materials and methods

### Cell lines and cell culture

Huh7 and Hep3B cells were obtained from Riken Cell Bank (Tsukuba, Japan). HEK-293 T cell lines were purchased from the American Type Culture Collection (Manassas, VA, USA). The MHCC-LM3 and MHCC-97H cell lines were obtained from the Liver Cancer Institute, Zhongshan Hospital of Fudan University (Shanghai, China). The HCC-LY10 cell line was established in our laboratory. The HCC cell lines used in this study were cultured in Dulbecco’s modified Eagle’s medium (DMEM) (Gibco) containing 10% heat-inactivated fetal bovine serum (Gibco) and incubated at 37°C in a humidified atmosphere with 5% CO_2_. All of the cell lines were authenticated and characterized by the suppliers. Cells were used within 6 months of resuscitation. These cell lines were mycoplasma-free and routinely authenticated by quality examinations of morphology and growth profile.

### Lentivirus production and cell transduction

The PSMD9 lentiviral overexpression plasmid and shRNA plasmid were supplied by the CCSB-Broad Lentiviral Expression Library and Human TRC shRNA Library. The EGFR plasmid was maintained in our laboratory [14]. The plasmid was sequenced from the 5′ and 3′ ends to confirm its sequence. The target sequences are listed in Supplementary Table 1.

Viral packaging was performed in HEK-293 T cells after cotransfection of the PSMD9 overexpression or shRNA plasmid with the packaging plasmid psPAX2 and the envelope plasmid pMD2.G (Addgene) using Lipofectamine 2000 (Invitrogen). The viruses were harvested at 72 h after transfection, and the viral titers were determined. HCC cells were transduced with 1 × 10^6^ recombinant lentivirus-transducing units in the presence of 6 μg/ml polybrene (Sigma).

### Quantitative real-time RT‒PCR (qRT‒PCR)

Total RNA extraction, reverse transcription, and qRT‒PCR analyses were performed as previously described using an ABI Prism 7500 System (Applied Biosystems, Carlsbad, CA, USA) with SYBR® *Premix Ex Taq* (Takara, Dalian, China). The mRNA levels were normalized to those of the housekeeping gene GAPDH. Sequences of primers are listed in Supplementary Table 2.

### Western blotting

Proteins in whole cell lysates were separated by SDS‒polyacrylamide gel electrophoresis and transferred onto PVDF membranes (Millipore). The membranes were incubated overnight with primary antibodies at 4 °C and then with secondary antibodies conjugated to horseradish peroxidase (HRP) for 1 h at room temperature. The immunoreactive blots were visualized using an enhanced chemiluminescence reagent (Pierce, Rockford, IL, USA). β-Actin was used as a loading control. Information of the antibodies is listed in Supplementary Table 3.

### Cell proliferation and colony formation assays

Cell proliferation was measured by the Cell Counting Kit-8 (CCK-8) (Bimake, USA) according to the manufacturer’s instructions. A cell proliferation EdU image Kit (Abbkine, Wuhan, China) was used for EdU staining following the manufacturer's protocols. The cells were observed and photographed with a fluorescence microscope after EdU staining. For colony formation assays, 1,000 cells were plated in each well of a 6-well plate and incubated at 37 °C for 2 weeks. Colonies were fixed with 4% phosphate-buffered formalin (pH 7.4) and subjected to Giemsa staining for 15 min. Three independent experiments were performed for each assay.

### Migration and invasion assays

Cells were seeded in the upper chamber of a transwell (8-μm pore size) or in a Matrigel-coated transwell (BD Biosciences, NJ) in serum free media. The lower chamber contained DMEM supplemented with 10% fetal bovine serum as a chemoattractant. After incubating for 24 or 48 h, the nonmigrated or noninvaded cells were gently removed from the upper chamber using a cotton swab. The cells were fixed with formalin and stained with Giemsa solution. The number of cells in five randomly chosen fields of view was counted under a microscope.

### Flow cytometry analysis

For cell cycle analysis, cells were washed twice with cold PBS, fixed in 70% cold ethanol and incubated overnight. Before analysis, the cells were stained with a solution containing 10 mg/ml RNAase and 400 mg/ml propidium iodide (PI) and incubated for 30 min at 37 °C. Finally, the cells were analyzed by flow cytometry.

For the apoptosis assay, the cells were harvested, washed, incubated with PE-conjugated Annexin V and 7-AAD, and incubated for 15 min at room temperature. The cells were analyzed by flow cytometry within 1 h. The results are the representative of 3 independent experiments with triplicate samples for each assay.

For the EGFR internalization assay, cells were trypsinized and collected after incubation with EGF at the indicated time points. After washing twice with cold PBS, nonpermeating cells were incubated with a PE-conjugated anti-EGFR antibody (BioLegend) for 30 min. After washing, the cells were immediately subjected to flow cytometry analysis (FlowJo 7.6.1).

### In vivo growth and metastasis assays

To assess the in vivo growth and metastasis of HCC cells, four- to six- week-old male BALB/C nude mice used; HCC cells were orthotopically inoculated into the left hepatic lobes of the mice with a microsyringe through an 8-mm transverse incision in the upper abdomen under anesthesia. A total of 2 × 10^6^ cells suspended in 40 μl of a mixture of serum-free DMEM/Matrigel (1:1 volume) (BD Biosciences, MA) were injected into each nude mouse. The mice were sacrificed at six weeks and the tissues were harvested and fixed with phosphate-buffered neutral formalin for at least 72 h. Metastases were identified by analyzing lung tissue sections followed by H&E staining.

For in vivo drug studies, six week old male BALB/C nude mice were injected subcutaneously with 2 × 10^6^ cells. When the tumors reached a volume of approximately 100 mm^3^ in size, the mice were randomized into 4 groups. The mice were treated with erlotinib (40 mg/kg, every 3 day) via oral gavage. The tumor dimensions were measured with Vernier calipers every 3 days, and tumor volume was calculated as follows: tumor volume = (length × width^2^)/2. On day 24, the mice were sacrificed, and the tumors were excised and fixed with 4% phosphate-buffered neutral formalin. All of the experiments were approved by the Renji Hospital Institutional Animal Care (RT2022-122u) and Use Committee and performed in accordance with the Institutional Guide for the Care and Use of Laboratory Animals.

### Immunofluorescence analysis via confocal imaging

Briefly, cells were grown on Lab-Tek chamber slides (Nunc), fixed with 4% paraformaldehyde in PBS for 30 min, and permeabilized with 0.1% Triton X-100 in PBS for 5 min. The slides were incubated with primary antibodies in blocking solution overnight at 4 °C in a humidified chamber. Subsequently, the glass slides were washed three times in PBS and incubated with Alexa Fluor 488-conjugated and Alexa Fluor 555-conjugated secondary antibodies and 4′, 6- diamidino-2-phenylindole (DAPI) in blocking solution for 30 min at 37 °C in a humidified chamber. Images were obtained with a Leica TCS SP8 confocal microscope (Leica, Microsystems). Information of the antibodies is listed in Supplementary Table S3.

### Coimmunoprecipitation (Co-IP) assay

The cells were harvested in RIPA (Upstate, Biotechnology) lysis buffer containing protease inhibitors for 40 min on ice and centrifuged at 12,000 × g for 10 min. Protein A/G agarose beads were incubated with anti-PSMD9 or anti-CBL antibody or negative control IgG overnight on an orbital shaker at 4 °C. The immune complex was precipitated with protein-A/G agarose, washed five times and analyzed by western blotting.

### Immunohistochemistry (IHC)

IHC assays were conducted as reported previously [15]. A total of 106 HCC tissues were obtained from Zhongshan Hospital of Fudan University. All samples were obtained with informed consent. The study was approved by the Ethics Committee of Renji Hospital, Shanghai Jiao Tong University School of Medicine (KY2023-084-B). Briefly, the sections were deparaffinized with xylene and rehydrated before being heated to just below boiling temperature at a subboiling temperature in sodium citrate buffer (pH 6.0) for 20 min in a microwave oven for antigen retrieval. After being washed with PBS three times, the samples were incubated with 3% hydrogen peroxide for 10 min to block endogenous peroxidase activity. The sections were then incubated overnight with primary antibodies at 4 °C. After being rinsed with PBS, the sections were incubated with horseradish peroxidase (HRP)-conjugated secondary antibody at 37 °C for 30 min and then incubated with diaminobenzidine solution. Finally, the nuclei were counterstained with Mayer’s hematoxylin.

### EGFR dimerization assay

Overnight serum-starved HCC cells were treated with EGF (25 ng/ml) at 4 °C for 1 h and then incubated with the 3 mM crosslinker BS3 (Thermo Fisher Scientific) at 4 °C for 20 min. The reaction was terminated by incubation with 250 mM glycine in PBS for 5 min. Samples were subjected to lysing and Western blotting.

### Statistical analysis

All the data are presented as the means ± standard deviations (SDs). Unpaired t test was used to compare the means between two groups, and one-way ANOVA was used to compare the means among multiple groups. The Kaplan‒Meier method was used to plot survival curves, which were compared by the log-rank test.* p* < 0.05 was considered to indicate statistical significance.

## Results

### Elevated PSMD9 expression in HCC is closely associated with increased tumor grade, metastasis and a poor prognosis

**Fig. 1:**
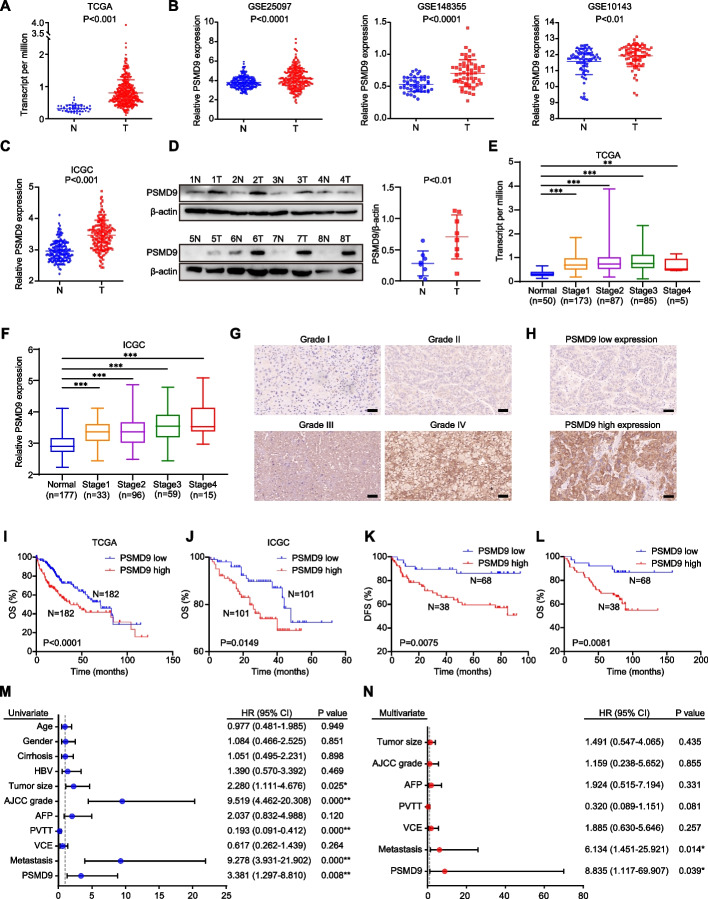
PSMD9 upregulation is associated with a poor prognosis in HCC patients. **A**-**C** The expression of PSMD9 in HCC tissues was compared with that in the corresponding noncancerous liver tissues in the TCGA datasets (*n* = 50) (**A**), the GSE10143, GSE25097 and GSE148355 (**B**) datasets and the ICGC-LIRI-JP dataset (**C**). **D** The expression of PSMD9 in HCC tissues was compared with that in the corresponding noncancerous liver tissues by Western blotting. **E**–**F** The expression of PSMD9 in noncancerous liver tissues and HCC tissues of different grades was analyzed in the TCGA (**E**) and ICGC-LIRI-JP cohorts (**F**). **G** Immunohistochemical analysis of PSMD9 expression in HCC samples. Representative images are shown. **H** Representative images of samples with high and low PSMD9 expression. **I**-**J** Overall survival analysis of HCC patients in the TCGA cohort (**I**) and the ICGC-LIRI-JP cohort (**J**) stratified by the PSMD9 expression. **K**-**L** Overall and disease free survival analysis of 106 HCC patients stratified by the PSMD9 expression level. **M**–**N** Univariate and multivariate Cox proportional hazards analyses were conducted to evaluate the HR of PSMD9 in terms of the overall survival of patients with HCC. *p < 0.05; **p < 0.01

We first analyzed the expression of PSMD9 in numerous cancers using The Cancer Genome Atlas (TCGA) data. We found that PSMD9 was highly expressed in a variety of human tumors, including HCC (Supplementary Figure S1A, Fig. 1A). We further confirmed the difference in PSMD9 expression between adjacent cancer tissue and cancer tissue by analyses of the Gene Expression Omnibus (GEO) and the JP Project from the International Cancer Genome Consortium (ICGC-LIRI-JP) (Fig. 1B-C). Moreover, PSMD9 protein levels were upregulated in HCC tissues compared with noncancerous tissues according to the Western blotting results (Fig. 1D). Furthermore, PSMD9 expression was upregulated in patients with nodal metastasis patients compared with patients without nodal metastasis in the TCGA cohorts (Supplementary Figure S1B).

We next investigated the clinical significance of PSMD9 in HCC patients. We found that the expression of PSMD9 was significantly correlated with the malignancy grade and metastasis in HCC patients in the TCGA and ICGC-LIRI-JP cohorts (Fig. 1E-F). We next assessed the expression of PSMD9 in HCC patients using IHC. The clinicopathological features of the patients with HCC (106 patients) were shown in Supplementary Table S4.We found that high PSMD9 expression positively correlated with high tumor grade in HCC patients (Fig. 1G). According to the IHC results, the patients were divided into two groups based on the expression of PSMD9 (Fig. 1H). We found that PSMD9 expression was positively associated with tumor size, American Joint Committee on Cancer (AJCC) grade and metastasis (Table 1). However, there was no correlation between PSMD9 expression and other clinicopathological factors, including gender, age, cirrhosis status, serum alpha-fetoprotein (AFP) level, HBV positivity status, portal vein tumor thrombus (PVTT) and vessel carcinoma embolus (VCE) (Table 1). Kaplan–Meier survival analysis revealed that high PSMD9 expression was associated with significantly shorter overall survival (OS) time and disease-free survival (DFS) time than low PSMD9 expression according to TCGA, ICGC and GEO data (Fig. 1I-J, Supplementary Figure S1C). The results were also confirmed using our cohort (Fig. 1K-L). Furthermore, univariate and multivariate Cox proportional hazard analyses suggested that high PSMD9 expression was associated with worse survival in HCC patients than low PSMD9 expression in HCC patients (*p* < 0.05; Fig. 1M-N). In addition, high expression of PSMD9 was associated with shorter OS times in patients with various types of tumors according to TCGA data (Supplementary Figure S2). Taken together, these findings indicate that high expression of PSMD9 is associated with a poor prognosis in HCC patients and that PSMD9 might play an important role in promoting the malignant progression of HCC.

**Table 1:** Correlation between PSMD9 levels in HCC patients and their clinicopathological characteristics

Clinicopathological features	Number	Low expression N (%)		High expression N (%)	*p* value
**Age**					
55	50	24(48.0)		26(52)	0.014*
≥ 55	56	14(25.0)		42(75.0)	
**Gender**					
Male	82	31(37.8)		51 (62.2)	0.438
Female	24	7(29.2)		17(70.8)	
**Tumor size**					
≤ 5 cm	65	29(44.6)		36(55.4)	0.010**
> 5 cm	40	8(20.0)		32(80.0)	
**AJCC grade**					
I- II	79	33(41.8)		46(58.2)	0.030*
III- IV	27	5(18.5)		22(81.5)	
**Cirrhosis**					
Negative	32	10(31.2)		22(68.8)	0.516
Positive	74	28(37.8)		46(62.2)	
**AFP (ng/mL)**					
≤ 20	33	14(42.4)		19(57.6)	0.345
> 20	70	23(32.9)		47(67.1)	
**HBV**					
Negative	17	4(23.5)		13(76.5)	0.248
Positive	89	34(38.2)		55(61.8)	
**Portal vein tumor thrombus (PVTT)**					
Negative	93		36(38.7)	57(61.3)	0.100
Positive	13		2(15.4)	11(84.6)	
**Vessel carcinoma embolus (VCE)**					
Negative	33		15(45.5)	18(54.5)	0.166
Positive	73		23(31.5)	50(68.5)	
**Metastasis**					
Negative	88		35(39.8)	53(60.2)	0.036*
Positive	17		2(11.8)	15(88.2)	

### PSMD9 promotes HCC cell proliferation

**Fig. 2:**
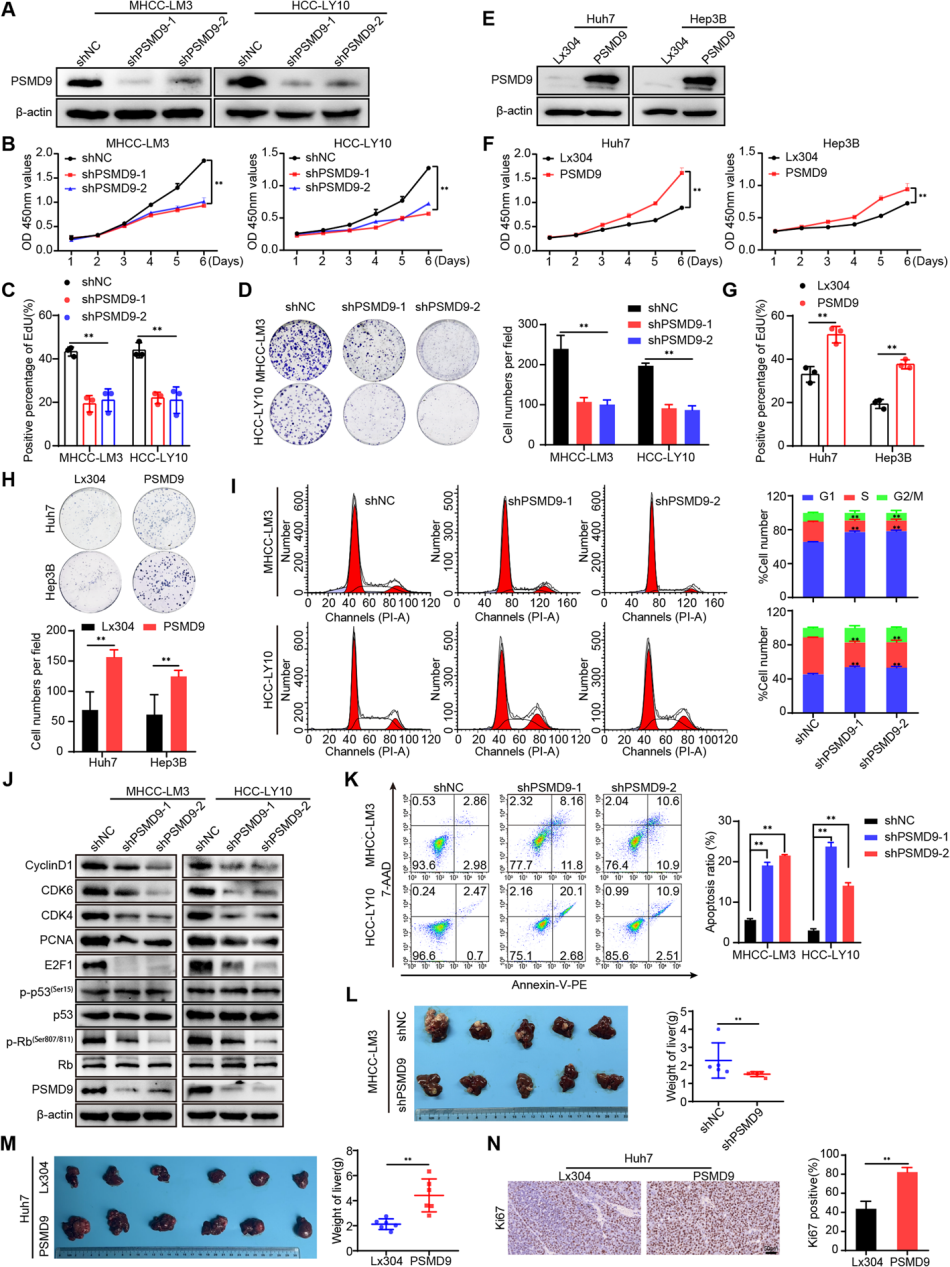
PSMD9 promotes HCC cell proliferation. **A** The expression levels of PSMD9 in PSMD9-knockdown HCC cells were determined by Western blotting. **B**-**D** The effect of PSMD9 knockdown on HCC cell proliferation was assessed by a CCK-8 assay (**B**), an EdU assay (**C**) and a colony formation assay (**D**). **E** The expression levels of PSMD9 in PSMD9-overexpressing HCC cells were determined by Western blotting. **F**–**H** The effect of PSMD9 overexpression on HCC cell proliferation was assessed by a CCK-8 assay (**F**), an EdU assay (**G**) and a colony formation assay (**H**). **I** The cell cycle distribution of cells was analyzed by flow cytometry. **J** The expression of cell cycle-related genes was detected by Western blotting. **K** Apoptosis was analyzed by flow cytometry. **L** Liver tissues from animals bearing xenografts from MHCC-LM3 cells with stable PSMD9 knockdown. The dot plots show the results of the quantitative analysis of liver weight. **M** Liver tissues from animals bearing xenografts from Huh7 cells with stable PSMD9 overexpression. The dot plots show the results of the quantitative analysis of liver weight. **N** Ki67 expression in xenograft tissues from Huh7 PSMD9-overexpressing cells was evaluated by IHC. Bar = 50 μm. *p < 0.05; **p < 0.01

To verify the function of PSMD9 in HCC, we first examined the expression of PSMD9 in HCC cell lines. We found that PSMD9 expression was high in HCC-LY10 and MHCC-LM3 cells and that PSMD9 expression was low in Huh7 and Hep3B cells (Supplementary Figure S3). The HCC-LY10 and MHCC-LM3 cell lines were subjected to PSMD9 knockdown via shRNA in subsequent experiments (Fig. 2A, supplementary Figure S4A). Our results showed that PSMD9 knockdown inhibited HCC cell proliferation and decreased the proportion of EdU-positive cells (Fig. 2B-D, supplementary Figure S4B). Conversely, exogenous expression of PSMD9 markedly promoted cell growth and colony formation ability (Fig. 2E-H, supplementary Figure S4C-D).

To further investigate the mechanism by which PSMD9 affects HCC proliferation, we determined the cell cycle distributions of HCC-LY10 and HCC-LM3 cells by flow cytometry. Our results showed that knockdown of PSMD9 increased the proportion of cells entering the G1 phase and decreased the proportion of cells entering the S phase, indicating that knockdown of PSMD9 induces cell cycle arrest at the G1 phase in HCC-LY10 and HCC-LM3 cells (Fig. 2I). We next detected the expression of cell cycle regulators. The results showed that the levels of CDK4, CDK6, cyclin D1, p-Rb, E2F1 and PCNA were drastically lower in PSMD9-knockdown cells than in control cells (Fig. 2J). Conversely, the expression of CDK4, CDK6, cyclin D1, p-Rb, E2F1 and PCNA was increased in PSMD9-overexpressing HCC cells(supplementary Figure S4E). However, expression of p53 was not affected in the PSMD9-knockdown and PSMD9-overexpressing HCC cells (Fig. 2J, supplementary Figure S4E). Furthermore, we found that knockdown of PSMD9 induced apoptosis in HCC cells (Fig. 2K). Conversely, overexpression of PSMD9 inhibited apoptosis in HCC cells (supplementary Figure S4F). Therefore, these results suggest that PSMD9 promotes the proliferation of HCC cells and inhibits their apoptosis.

Next, we assessed the effect of PSMD9 on the tumorigenicity of HCC cells in vivo by using an orthotopic liver tumor model in nude mice. As shown in Fig. 2L, MHCC-LM3 tumors derived from shPSMD9 group cells weighed less than those derived from shNC group cells. In contrast, compared with the control cells, Huh7 cells overexpressing PSMD9 promoted tumor growth, as determined by the liver weight (Fig. 2M). Furthermore, the expression of Ki-67 was significantly increased in HCC cells overexpressing PSMD9 (Fig. 2N). Taken together, these results provide strong evidence that PSMD9 promotes the tumorigenesis.

### PSMD9 promotes HCC cell migration, invasion and metastasis

**Fig. 3:**
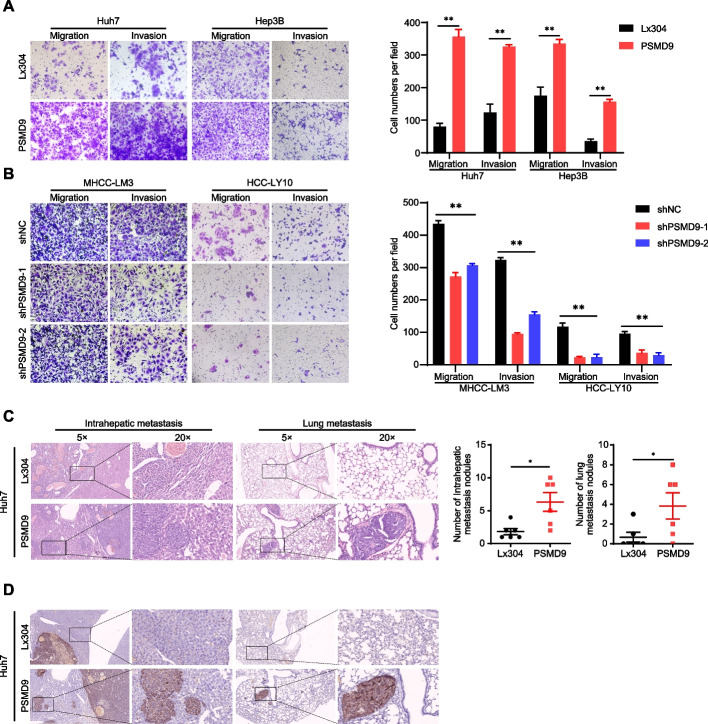
PSMD9 promotes HCC cell invasion and metastasis. **A**-**B** The effects of PSMD9 overexpression (**A**) and knockdown (**B**) on HCC cell migration and invasion were assessed by transwell assays. **C** Representative images of intrahepatic nodules and lung nodules formed by PSMD9-overexpressing Huh7 cells and control cells are shown. The numbers of intrahepatic metastatic nodules and lung metastatic nodules are shown in the right panel. Bar = 50 μm. **D** IHC analysis of metastasis using human-specific anti-mitochondria antibodies. *p < 0.05; **p < 0.01

Cancer cell migration and invasion have been identified as key events in cancer development. Therefore, we examined the effect of PSMD9 on HCC cell migration and invasion. The results showed that the overexpression of PSMD9 promoted HCC cell migration and invasion, whereas silencing PSMD9 resulted in decreased cell migration and invasion (Fig. 3A-B).

To further clarify the role of PSMD9 in HCC metastasis in vivo, PSMD9-overexpressing cells were orthotopically inoculated into the left hepatic lobe of mice via a microsyringe. Histological examination of lung and liver tissues indicated that the number of intrahepatic and lung metastasis nodules was significantly greater in the PSMD9 overexpression group than in the control group (Fig. 3C). Metastasis was confirmed by anti-human mitochondria antibody staining, which is used to detect human cells in xenograft models (Fig. 3D). Taken together, these findings suggest that PSMD9 promotes HCC metastasis.

### PSMD9 promotes EGFR expression through inhibition of its ubiquitination

**Fig. 4:**
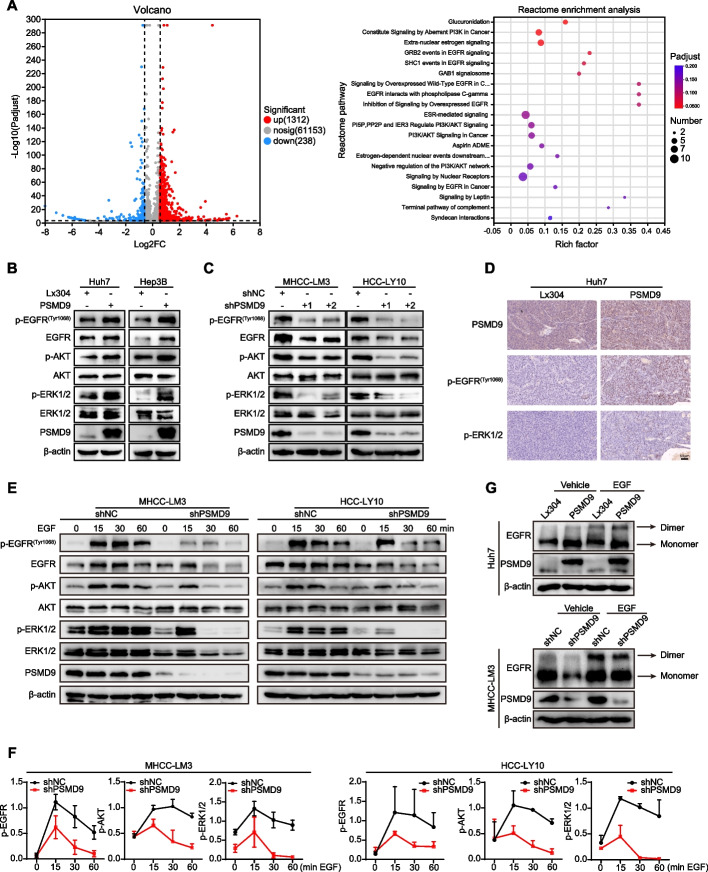
PSMD9 promotes EGFR, ERK1/2, and Akt phosphorylation. **A** Ectopic overexpression of PSMD9 activated the EGFR signaling pathway according to Reactome enrichment analysis. **B**-**C** The expression of p-EGFR, EGFR, p-ERK1/2, ERK1/2, p-Akt and Akt in PSMD9-overexpressing Huh7 and Hep3B cells (**B**) and PSMD9-knockdown HCC-LY10 and MHCC-LM3 cells (**C**) was detected by Western blotting. **D** The expression of p-EGFR and p-ERK1/2 in xenograft tumor tissues from Huh7-PSMD9 and control cells was detected by IHC. **E** Knocking down PSMD9 promoted p-EGFR, p-ERK1/2 and p-Akt degradation after EGF stimulation. **F** Densitometric analysis of the expression levels of p-EGFR, p-ERK1/2 and p-Akt. **G** The dimer and monomer forms of EGFR were evaluated in PSMD9-overexpressing and PSMD9-knockdown cells

To explore the mechanism of PSMD9 in HCC, RNA sequencing was performed in PSMD9-overexpressing cells. Many differentially expressed genes (DEGs) were identified in cells based on RNA-seq, and DEGs were further subjected to Reactome enrichment analysis. The results of Reactome functional enrichment analysis showed that PSMD9 can regulate EGFR signaling in HCC cells (Fig. 4A). Therefore, we next detected the expression of EGFR in PSMD9-overexpressing and PSMD9-knockdown HCC cells. Our results showed that the overexpression of PSMD9 increased the phosphorylation of EGFR in HCC cells (Fig. 4B). In contrast, EGFR phosphorylation was inhibited in PSMD9-knockdown HCC cells (Fig. 4C). EGFR triggers several signal transduction cascades, including those of Raf1-extracellular signal-regulated kinase (ERK) and PI3K-Akt. Therefore, we detected the expression of ERK1/2 and Akt in HCC cells. Our results showed that overexpression of PSMD9 promoted the phosphorylation of ERK1/2 and Akt in HCC cells (Fig. 4B). Conversely, PSMD9 knockdown inhibited the phosphorylation of ERK1/2 and Akt in HCC cells (Fig. 4C). Furthermore, phosphorylated EGFR and its downstream signaling molecule phosphorylated ERK1/2 were activated in murine xenografts from PSMD9-overexpressing Huh7 cells (Fig. 4D). To determine whether PSMD9 affects EGF-induced EGFR degradation, we detected the expression of EGFR and its downstream signaling proteins in PSMD9-knockdown HCC cells treated with EGF. Our results showed that knocking down PSMD9 inhibited the phosphorylation of EGFR in HCC cells treated with EGF. Moreover, the activation of ERK1/2 and Akt was also altered in PSMD9-knockdown HCC cells treated with EGF (Fig. 4E-F). Therefore, these results indicate that PSMD9 regulates EGFR signaling in HCC cells.

EGFR is a cell surface protein that binds to epidermal growth factor. Binding of the protein to a ligand induces receptor dimerization and tyrosine autophosphorylation and leads to cell proliferation [16]. Therefore, crosslinking experiments were performed to examine the effects of PSMD9 on EGFR dimerization and EGF-induced dimerization of EGFR, and we found that forced expression of PSMD9 increased EGFR dimerization and EGF-induced EGFR dimerization. Conversely, PSMD9 knockdown inhibited EGFR dimerization and EGF-induced EGFR dimerization (Fig. 4G).

**Fig. 5:**
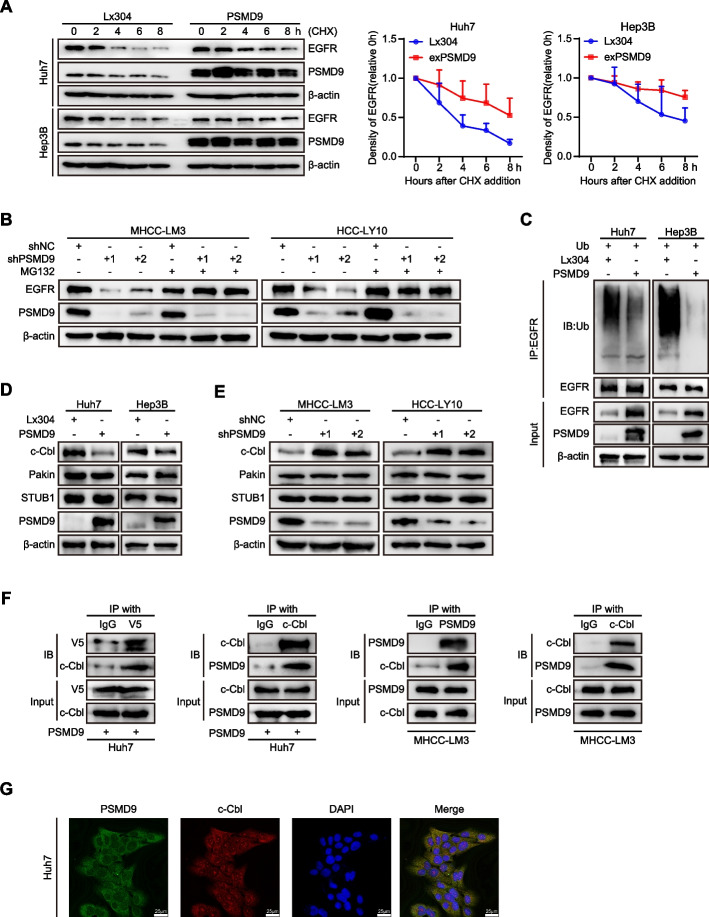
PSMD9 binds c-Cbl and is associated with EGFR ubiquitination. **A** Expression of EGFR in PSMD9-overexpressing HCC cells treated with cycloheximide (CHX) at the indicated time points. **B** Expression of EGFR in PSMD9-knockdown HCC cells treated with MG132 for 6 h. **C** Lysates of PSMD9-overexpressing and control Huh7 and Hep3B cells were immunoprecipitated with an anti-EGFR antibody, and the immunocomplexes were immunoblotted with antibodies against ubiquitinated proteins. **D**-**E** The expression of c-Cbl, Pakin and STUB1 in PSMD9-overexpressing (**D**) and PSMD9-knockdown (**E**) HCC cells was detected by Western blotting. **F** Co-IP and Western blotting showed that PSMD9 and c-Cbl bind to each other. **G** The protein expression of PSMD9 and c-Cbl in Huh7 cells was determined by immunofluorescence assays. **p < 0.01

In addition, we found that PSMD9 did not affect the expression of EGFR mRNA in HCC cells (Supplementary Figure S5A). These results indicate that PSMD9 promotes EGFR expression through posttranscriptional regulation. Next, PSMD9-overexpressing or control HCC cells were treated with cycloheximide (50 μg/ml), which blocks de novo protein synthesis. Our results showed that the rate of EGFR protein degradation was significantly lower in PSMD9-overexpressing cells than in control cells (Fig. 5A). The half-life of the EGFR protein in the cells was extended (from 3 to 8 h in Huh7 cells and from 6 to 12 h in Hep3B cells) as a consequence of PSMD9 overexpression (Fig. 5A). These results suggest that the overexpression of PSMD9 increases the stability of the EGFR protein in HCC cells.

To investigate the involvement of the ubiquitin‒proteasome pathway in the proteolytic degradation of EGFR, we applied the proteasomal inhibitor MG132 to PSMD9-knockdown HCC cells. Our results showed that the downregulation of EGFR caused by PSMD9 knockdown was blocked by MG132 treatment (Fig. 5B). Furthermore, overexpression of PSMD9 significantly reduced the ubiquitination of EGFR in an in vitro ubiquitination assay (Fig. 5C). Therefore, these results indicated that PSMD9 increases the stability of EGFR by reducing its ubiquitination.

### PSMD9 interacts with c-Cbl and stabilizes EGFR

To determine whether E3 ubiquitin ligases are involved in the regulation of EGFR ubiquitination by PSMD9, we next examined the expression of the ubiquitin ligases of EGFR (STUB1, Parkin and c-Cbl) in PSMD9-overexpressing and PSMD9-knockdown HCC cells [17–19]. Our results showed that overexpression of PSMD9 inhibited the expression of c-Cbl in HCC cells. Conversely, PSMD9 knockdown increased c-Cbl expression in HCC cells. However, the Pakin and STUB1 levels were not significantly influenced by PSMD9 in HCC cells (Fig. 5D-E). Next, to investigate whether PSMD9 binds specifically to c-Cbl in HCC cells, we carried out co-IP and found that PSMD9 interacts with c-Cbl directly (Fig. 5F, supplementary Figure S5B). Furthermore, immunofluorescence staining revealed that PSMD9 and c-Cbl were colocalized in HCC cells (Fig. 5G). Taken together, these results indicate that PSMD9 interacts with c-Cbl and inhibits its expression thus decreasing the level of EGFR ubiquitination to increase EGFR stability in HCC cells.

### PSMD9 influences EGFR endocytosis and degradation

**Fig. 6:**
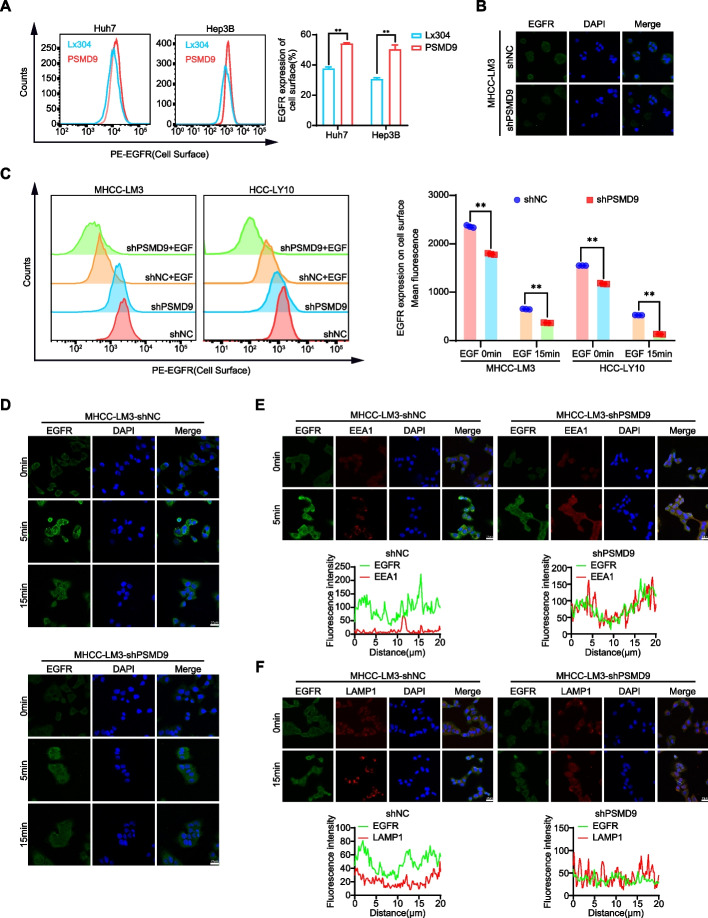
PSMD9 influences EGFR endosomal trafficking. **A** EGFR expression on the cell surface was assessed by flow cytometry. **B** The expression of EGFR in PSMD9-knockdown MHCC-LM3 cells was assessed by immunofluorescence. **C** EGFR expression on the cell surface of PSMD9-knockdown MHCC-LM3 cells was assessed by flow cytometry in the presence of EGF. **D** The expression of EGFR in the presence of EGF for the indicated time periods was assessed by immunofluorescence. **E** PSMD9-knockdown MHCC-LM3 cells incubated with EGF for the indicated time periods were subjected to an immunofluorescence assay. Antibodies against EGFR and EEA1 were used. **F** PSMD9-knockdown MHCC-LM3 cells incubated with EGF for the indicated time periods were subjected to an immunofluorescence assay. Antibodies against EGFR and LAMP1 were used. **p < 0.01

EGFR endocytosis and degradation are regulated by the ubiquitination of EGFR [20]. Therefore, we first detected the cell surface expression of EGFR in the PSMD9-overexpressing HCC cells. We found that the overexpression of PSMD9 promoted the cell surface expression of EGFR (Fig. 6A). Knockdown of PSMD9 inhibited the expression of EGFR in HCC cells as determined by immunofluorescence (Fig. 6B). Next, we detected the cell surface expression of EGFR in the PSMD9-knockdown HCC cells treated with EGF. The results showed that knockdown of PSMD9 decreased the cell surface expression of EGFR compared with that in control cells (Fig. 6C-D). Furthermore, PSMD9 knockdown promoted the colocalization of EGFR with the lysosomal degradation markers EEA1 (a marker of the early endosomal stage) and LAMP1 (a lysosomal marker) (Fig. 6E-F). Taken together, these results indicate that PSMD9 inhibits c-Cbl expression and subsequently decreases EGFR ubiquitination and endocytosis from the cell membrane and suppresses ERK1/2 and Akt activation, which contributes to HCC progression.

### PSMD9 promotes HCC cell proliferation, migration and invasion through the EGFR pathway

**Fig. 7:**
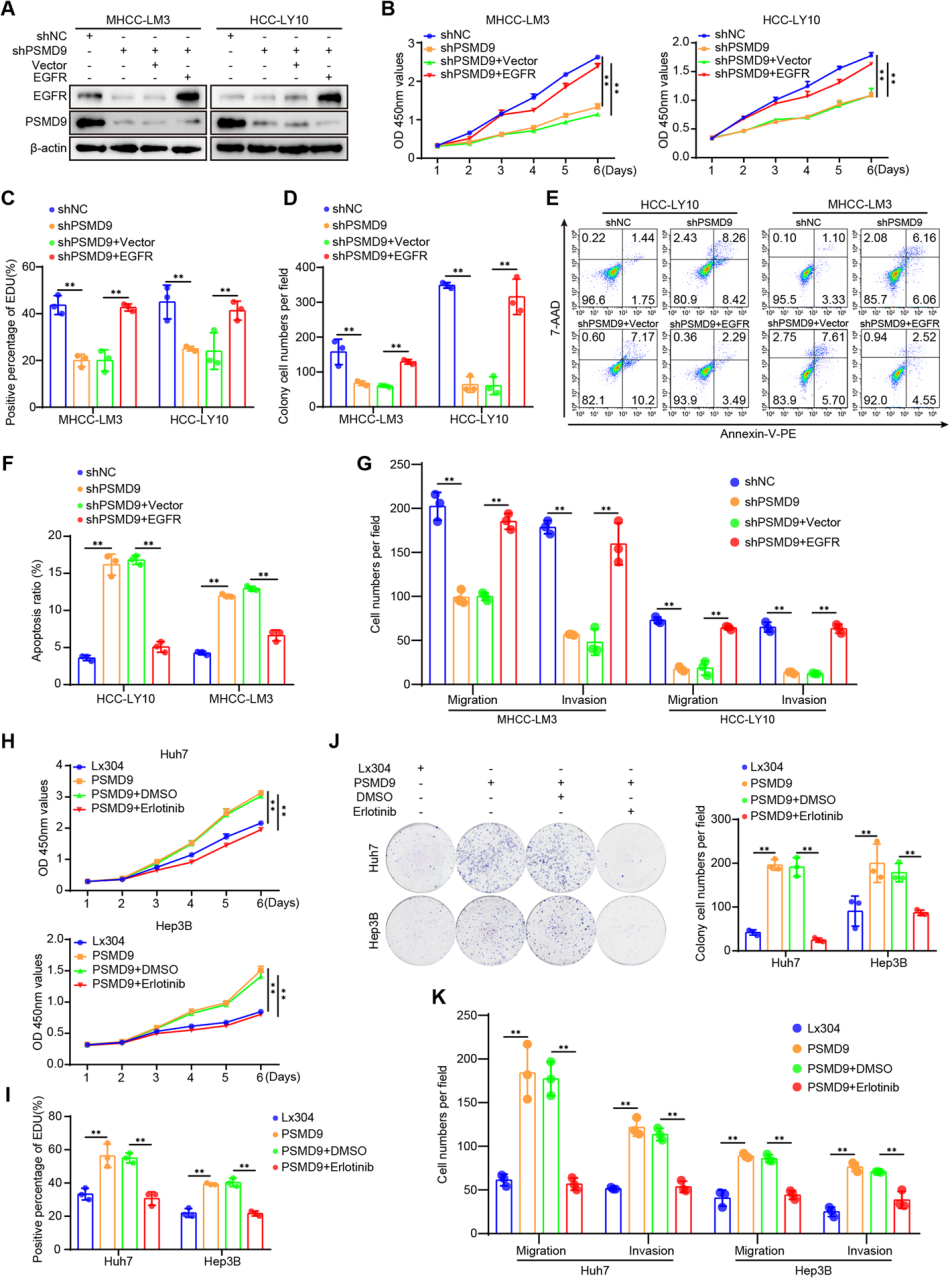
PSMD9 regulates HCC cell functions via the EGFR signaling. **A** PSMD9-knockdown HCC cells were transfected with EGFR, and the expression of EGFR and PSMD9 was detected by Western blotting. **B**-**E** PSMD9-knockdown HCC cells were transfected with EGFR as indicated, and cell proliferation, apoptosis, migration and invasion were evaluated by CCK-8 assay (**B**), EdU assay (**C**), colony formation (**D**), flow cytometry (**E**–**F**) and Transwell assays (**G**). **H**–**K** PSMD9-overexpressing HCC cells were treated with erlotinib or DMSO as indicated, and cell proliferation, migration and invasion were evaluated by CCK-8 assay (**H**), EdU assay (**I**),colony formation (**J**), and Transwell assays (**K**). *p < 0.05; **p < 0.01

To confirm the role of EGFR in PSMD9-mediated HCC proliferation and invasion, we assessed the effect of EGFR expression on the cell proliferation and invasion of PSMD9-knockdown cells (Fig. 7A). The results showed that the PSMD9 knockdown-induced suppression of cell proliferation, migration and invasion could be reversed by overexpressing EGFR in HCC cells (Fig. 7B-G, supplementary Figure S6A-S6C). PSMD9 knockdown-induced apoptosis also was reversed by overexpressing EGFR in HCC cells (Fig. 7E-F). In addition, the EGFR inhibitor erlotinib was used to treat PSMD9-overexpressing HCC cells. Our results showed that the PSMD9 overexpression-induced promotion of cell proliferation, migration and invasion could be attenuated by erlotinib in HCC cells (Fig. 7H-K, supplementary Figure S6D-S6E). In addition, PSMD9 overexpression-induced cell proliferation, migration, and invasion were reversed by EGFR shRNAs (Supplementary Figure S7A-F).Therefore, these results suggested that PSMD9 promotes cell proliferation and invasion through the EGFR pathway in HCC cells.

### Knockdown of PSMD9 sensitizes HCC cells to erlotinib

**Fig. 8:**
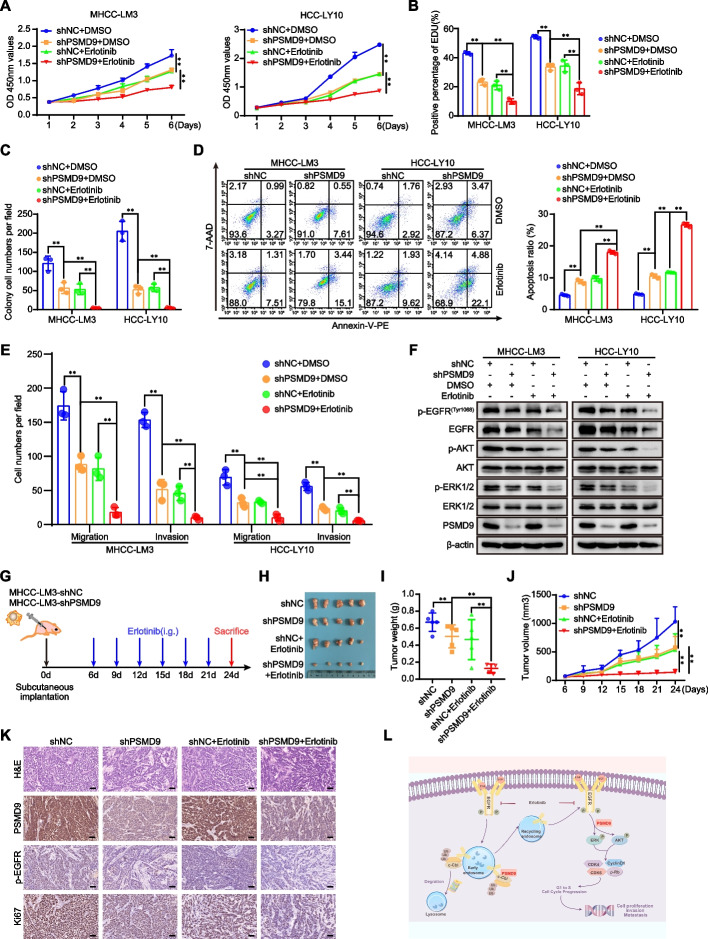
Knockdown of PSMD9 sensitizes HCC cells to erlotinib. **A**-**E** PSMD9 knockdown or control cells were incubated with erlotinib. Cell proliferation was detected by a CCK-8 assay (**A**), an EdU incorporation assay (**B**) and a colony formation assay (**C**). Apoptosis was detected by flow cytometry (**D**). Migration and invasion were detected by transwell assays (**E**). The expression of p-EGFR, EGFR, p-Akt, Akt, p-ERK1/2, ERK1/2 and PSMD9 was detected by western blotting (**F**). **G**-**K** In vivo xenograft tumorformation assays were performed using stably PSMD9 knockdown or control MHCC-LM3 cells (2 × 10^6^) subcutaneously injected into the right posterior flanks of 6-week-old male BALB/C nude mice, followed by treatment with vehicle or erlotinib (40 mg/kg/d) when the tumors reached a volume of approximately 100 mm^3^ in size. Schematic of the experimental design (**G**). On day 24, the mice were sacrificed, and the tumors were photographed (**H**). Tumor weights were measured and plotted (**I**). Tumor growth was measured every 3 days (**J**). The expression of Ki67, PSMD9 and p-EGFR in xenograft tissues was evaluated by IHC (**K**). **L** Model of the mechanisms of action of PSMD9 in HCC. PSMD9 interacts with c-Cbl, stabilizes EGFR, decreases EGFR ubiquitination, influences EGFR endosomal trafficking and degradation and subsequently activates downstream signaling and promotes HCC progression. Bar = 50 μm. *p < 0.05; **p < 0.01

According to the results mentioned above, we asked whether PSMD9 can be regarded as a potential target for HCC therapy. Erlotinib combined with sorafenib did not improve survival in patients with advanced HCC [4].To confirm whether the knockdown of PSMD9 enhances the potential effect of erlotinib on HCC cells, HCC cells with PSMD9 knockdown were incubated with erlotinib. As shown in Fig. 8, PSMD9 knockdown sensitized HCC cells to erlotinib. Erlotinib treatment combined with PSMD9 knockdown had synergistic inhibitory effects on cell proliferation, migration and invasion (Fig. 8A-E, supplementary Figure S8A-S8C). Knockdown of PSMD9 enhanced erlotinib-induced cell apoptosis (Fig. 8D). Long-term colony formation assays revealed that compared with control cells, PSMD9-knockdown cells were sensitive to erlotinib treatment (Fig. 8C, supplementary Figure S8B). In addition, erlotinib treatment combined with PSMD9 knockdown synergistically inhibited the expression of EGFR and the phosphorylation of ERK1/2 and Akt in HCC cells (Fig. 8F). Therefore, these data indicate that the loss of PSMD9 sensitizes HCC cells to erlotinib. We next evaluated the synergistic effect of this combination treatment in vivo. We found that PSMD9 knockdown increased the sensitivity of HCC cells to erlotinib treatment. PSMD9 knockdown combined with erlotinib treatment reduced the overall tumor volume and mass (Fig. 8G-J). In addition, the positive expression of Ki67 and EGFR was decreased significantly, as indicated by the immunohistochemistry results (Fig. 8K).

## Discussion

Several studies have reported that PSMD9 is correlated with the development of many tumor types. For example, low PSMD9 expression is associated with relative tumor radiosensitivity in breast cancer [11]. PSMD9 expression is correlated with recurrence after radiotherapy in patients with cervical cancer [10]. Although the role of PSMD9 in many tumor types has been studied, there are few reports about the role and molecular mechanism of PSMD9 in HCC. In the present work, we found that the expression of PSMD9 was significantly upregulated in HCC tissues. Furthermore, the expression of PSMD9 was significantly correlated with the malignancy grade, metastasis status and prognosis of HCC patient. We found that PSMD9 overexpression promoted cell growth and metastasis. Furthermore, PSMD9 knockdown sensitized HCC cells to erlotinib. Therefore, PSMD9 may be a therapeutic target or may be used to guide therapy. At present, proteasome inhibitors are an important class of drugs for the treatment of multiple myeloma [21]. Numerous preclinical experiments have demonstrated that combination treatment with proteasome inhibitors can markedly increase the therapeutic effects on cancer cells [22]. Highly selective specific proteasome inhibitors are beneficial for the treatment of solid tumors.

The main function of the proteasome is to regulate cell fate. Numerous studies have revealed that inhibition or knockdown of proteasomal subunits causes cell death in cancer cells. PSMD2 knockdown inhibits breast cancer cell proliferation and arrests the cell cycle [23]. Silencing PSMD4 regulates cell cycle arrest by modulating PTEN/Akt signaling in HCC [9]. PSMD10 inhibition suppresses autophagy and induces HCC cell sensitivity to drugs [24]. In this study, our results revealed that cell cycle arrest induced by PSMD9 knockdown is associated with decreased expression of G1 phase related proteins, such as cyclin D1 and CDK4/6. Apoptosis was also observed in PSMD9-knockdown HCC cells. Activated EGFR/ERK signaling has been tightly linked to the expression of cyclin D1 [25]. We found that PSMD9 promotes EGFR expression in HCC cells. Therefore, these data indicate that PSMD9 plays an important role in HCC cell proliferation.

The EGFR signaling pathway has been shown to be involved in the pathogenesis of several malignancies, including HCC [26, 27]. EGFR is frequently mutated and/or overexpressed in different types of human cancers and the molecular target of multiple cancer therapies [28]. EGFR activation can accelerate intracellular signaling cascades, leading to the activation of downstream effectors, such as the PI3K/Akt, MAPK, Ras/Raf/Mek/Erk, JAK/STAT, and PLCγ1/PKC pathways [2]. ERK1/2 and Akt are thought to be downstream effectors of EGFR signaling. Activated ERK1/2 translocates to the nucleus to activate ternary complex factor (TCF) transcription factors, which bind to the cyclin D1 promoter to promote G1/S phase transition [29]. Activated Akt is associated with HCC progression [9, 30]. Our results revealed that PSMD9 promotes EGFR expression and EGFR dimerization, leading to the activation of ERK1/2 and Akt in HCC cells. Knockdown of PSMD9 suppresses EGF-induced phosphorylation of EGFR and dimerization of EGFR and also suppresses ERK1/2 activation. Activated EGFR undergoes internalization, degradation or recycling in the absence of ligand [31]. Ubiquitin serves as a sorting signal during the endocytosis of EGFR [32]. Recruitment of the E3 ubiquitin ligase c-Cbl to activated EGFR is a key event leading to receptor ubiquitylation. C-Cbl directly binds to Py1045 or indirectly binds to the pY1068/pY1086 residues of EGFR via the GRB2 adaptor protein [33]. Our previous research also revealed that sorting nexin 5 interacts with EGFR and influences endosomal trafficking and degradation of EGFR in HCC [14]. In this study, we found that PSMD9 inhibits c-Cbl expression and subsequently suppresses EGFR ubiquitination and endocytosis from the cell membrane. Furthermore, we found that the effect of PSMD9 on HCC cell proliferation, migration and invasion was reversed by the EGFR inhibitor erlotinib and EGFR shRNA. Therefore, these data indicated that PSMD9 promotes HCC cell proliferation and metastasis through the EGFR-mediated signaling pathway.

First-generation drugs, including erlotinib and gefitinib, are reversible inhibitors. Approximately 60% of patients with acquired resistance to EGFR TKIs (erlotinib, gefitinib, and afatinib) develop a new mutation within the drug target. The EGFR T790M mutation is as the most common mechanism of acquired resistance [34, 35]. The proteasome has become an attractive target for the treatment of many cancers. Several proteasome inhibitors have displayed remarkable antitumor effects. Many studies have shown that regulating the ubiquitin‒proteasome system has great potential as an approach for overcoming drug resistance [36]. Therefore, knockdown of PSMD9 or the discovery of PSMD9 inhibitors can promote the ubiquitination-mediated degradation of EGFR and Increase the sensitivity to EGFR TKIs.

In conclusion, our findings demonstrated that PSMD9 upregulation was associated with a poor prognosis in HCC patients. PSMD9 promotes HCC cell proliferation and metastasis via direct interaction with c-Cbl, subsequently decreasing EGFR ubiquitination and influencing EGFR endosomal trafficking and degradation (Fig. 8K). Our findings highlight the molecular mechanism of PSMD9 in HCC progression and provide valuable information for cancer prognosis evaluation and treatment.

## Supplementary Information

Supplementary Material 1.

## Abbreviations

HCC: Hepatocellular carcinoma
TCGA: The Cancer Genome Atlas
GEO: Gene Expression Omnibus
ICGC-LIRI-JP: JP Project from International Cancer Genome Consortium
EGFR: Epidermal growth factor receptor
RTKs: Receptor tyrosine kinases
Ub: Ubiquitin-proteasome
PI: Propidium iodide
CHX: Cycloheximide
IHC: Immunohistochemistry
Co-IP: Coimmunoprecipitation
EEA1: Early endosome antigen 1
LAMP1: Lysosomal Associated Membrane Protein 1
PVTT: Portal vein tumor thrombus
VCE: Vessel carcinoma embolus
c-Cbl: Cbl proto-oncogene C

## References

[1] Villanueva A. Hepatocellular Carcinoma. N Engl J Med. 2019;380:1450–62. 30970190 10.1056/NEJMra1713263

[2] Levantini E, Maroni G, Del Re M, Tenen DG. EGFR signaling pathway as therapeutic target in human cancers. Semin Cancer Biol. 2022;85:253–75. 35427766 10.1016/j.semcancer.2022.04.002

[3] Singh M, Jadhav HR. Targeting non-small cell lung cancer with small-molecule EGFR tyrosine kinase inhibitors. Drug Discov Today. 2018;23:745–53. 29031620 10.1016/j.drudis.2017.10.004

[4] Zhu AX, Rosmorduc O, Evans TR, Ross PJ, Santoro A, Carrilho FJ, et al. SEARCH: a phase III, randomized, double-blind, placebo-controlled trial of sorafenib plus erlotinib in patients with advanced hepatocellular carcinoma. J Clin Oncol. 2015;33:559–66. 25547503 10.1200/JCO.2013.53.7746

[5] Mani A, Gelmann EP. The ubiquitin-proteasome pathway and its role in cancer. J Clin Oncol. 2005;23:4776–89. 16034054 10.1200/JCO.2005.05.081

[6] Collins GA, Goldberg AL. The Logic of the 26S Proteasome. Cell. 2017;169:792–806. 28525752 10.1016/j.cell.2017.04.023 PMC5609836

[7] Majeed S, Aparnathi MK, Nixon KCJ, Venkatasubramanian V, Rahman F, Song L, et al. Targeting the Ubiquitin-Proteasome System Using the UBA1 Inhibitor TAK-243 is a Potential Therapeutic Strategy for Small-Cell Lung Cancer. Clin Cancer Res. 2022;28:1966–78. 35165102 10.1158/1078-0432.CCR-21-0344 PMC9365348

[8] Manasanch EE, Orlowski RZ. Proteasome inhibitors in cancer therapy. Nat Rev Clin Oncol. 2017;14:417–33. 28117417 10.1038/nrclinonc.2016.206 PMC5828026

[9] Jiang Z, Zhou Q, Ge C, Yang J, Li H, Chen T, et al. Rpn10 promotes tumor progression by regulating hypoxia-inducible factor 1 alpha through the PTEN/Akt signaling pathway in hepatocellular carcinoma. Cancer Lett. 2019;447:1–11. 30673593 10.1016/j.canlet.2019.01.020

[10] Koster F, Sauer L, Hoellen F, Ribbat-Idel J, Brautigam K, Rody A, et al. PSMD9 expression correlates with recurrence after radiotherapy in patients with cervical cancer. Oncol Lett. 2020;20:581–8. 32565983 10.3892/ol.2020.11622 PMC7285846

[11] Langlands FE, Dodwell D, Hanby AM, Horgan K, Millican-Slater RA, Speirs V, et al. PSMD9 expression predicts radiotherapy response in breast cancer. Mol Cancer. 2014;13:73. 24673853 10.1186/1476-4598-13-73 PMC4230020

[12] Zhao Y, Wen S, Li H, Pan CW, Wei Y, Huang T, et al. Enhancer RNA promotes resistance to radiotherapy in bone-metastatic prostate cancer by m(6)A modification. Theranostics. 2023;13:596–610. 36632223 10.7150/thno.78687 PMC9830431

[13] Ud Din Farooqee SB, Christie J, Venkatraman P. PSMD9 ribosomal protein network maintains nucleolar architecture and WT p53 levels. Biochem Biophys Res Commun. 2021;563:105–12 10.1016/j.bbrc.2021.05.004 34077860

[14] Zhou Q, Huang T, Jiang Z, Ge C, Chen X, Zhang L, et al. Upregulation of SNX5 predicts poor prognosis and promotes hepatocellular carcinoma progression by modulating the EGFR-ERK1/2 signaling pathway. Oncogene. 2020;39:2140–55. 31819169 10.1038/s41388-019-1131-9

[15] Zhou Q, Tian W, Jiang Z, Huang T, Ge C, Liu T, et al. A Positive Feedback Loop of AKR1C3-Mediated Activation of NF-kappaB and STAT3 Facilitates Proliferation and Metastasis in Hepatocellular Carcinoma. Cancer Res. 2021;81:1361–74. 33361392 10.1158/0008-5472.CAN-20-2480

[16] Sabbah DA, Hajjo R, Sweidan K. Review on Epidermal Growth Factor Receptor (EGFR) Structure, Signaling Pathways, Interactions, and Recent Updates of EGFR Inhibitors. Curr Top Med Chem. 2020;20:815–34. 32124699 10.2174/1568026620666200303123102

[17] Hou J, Deng Q, Zhou J, Zou J, Zhang Y, Tan P, et al. CSN6 controls the proliferation and metastasis of glioblastoma by CHIP-mediated degradation of EGFR. Oncogene. 2017;36:1134–44. 27546621 10.1038/onc.2016.280

[18] Lin DC, Xu L, Chen Y, Yan H, Hazawa M, Doan N, et al. Genomic and Functional Analysis of the E3 Ligase PARK2 in Glioma. Cancer Res. 2015;75:1815–27. 25877876 10.1158/0008-5472.CAN-14-1433 PMC4417379

[19] Levkowitz G, Waterman H, Ettenberg SA, Katz M, Tsygankov AY, Alroy I, et al. Ubiquitin ligase activity and tyrosine phosphorylation underlie suppression of growth factor signaling by c-Cbl/Sli-1. Mol Cell. 1999;4:1029–40. 10635327 10.1016/s1097-2765(00)80231-2

[20] Haglund K, Sigismund S, Polo S, Szymkiewicz I, Di Fiore PP, Dikic I. Multiple monoubiquitination of RTKs is sufficient for their endocytosis and degradation. Nat Cell Biol. 2003;5:461–6. 12717448 10.1038/ncb983

[21] Gandolfi S, Laubach JP, Hideshima T, Chauhan D, Anderson KC, Richardson PG. The proteasome and proteasome inhibitors in multiple myeloma. Cancer Metastasis Rev. 2017;36:561–84. 29196868 10.1007/s10555-017-9707-8

[22] Zhang L, Wu M, Su R, Zhang D, Yang G. The Efficacy and Mechanism of Proteasome Inhibitors in Solid Tumor Treatment. Recent Pat Anticancer Drug Discov. 2022;17:268–83. 34856915 10.2174/1574892816666211202154536

[23] Li Y, Huang J, Zeng B, Yang D, Sun J, Yin X, et al. PSMD2 regulates breast cancer cell proliferation and cell cycle progression by modulating p21 and p27 proteasomal degradation. Cancer Lett. 2018;430:109–22. 29777785 10.1016/j.canlet.2018.05.018

[24] Luo T, Fu J, Xu A, Su B, Ren Y, Li N, et al. PSMD10/gankyrin induces autophagy to promote tumor progression through cytoplasmic interaction with ATG7 and nuclear transactivation of ATG7 expression. Autophagy. 2016;12:1355–71. 25905985 10.1080/15548627.2015.1034405 PMC4968225

[25] Musgrove EA, Caldon CE, Barraclough J, Stone A, Sutherland RL. Cyclin D as a therapeutic target in cancer. Nat Rev Cancer. 2011;11:558–72. 21734724 10.1038/nrc3090

[26] Lanaya H, Natarajan A, Komposch K, Li L, Amberg N, Chen L, et al. EGFR has a tumour-promoting role in liver macrophages during hepatocellular carcinoma formation. Nat Cell Biol. 2014;16:972–7. 25173978 10.1038/ncb3031 PMC4183558

[27] Song S, Yu Z, You Y, Liu C, Xie X, Lv H, et al. EGFR/MET promotes hepatocellular carcinoma metastasis by stabilizing tumor cells and resisting to RTKs inhibitors in circulating tumor microemboli. Cell Death Dis. 2022;13:351. 35428350 10.1038/s41419-022-04796-8 PMC9012802

[28] Sigismund S, Avanzato D, Lanzetti L. Emerging functions of the EGFR in cancer. Mol Oncol. 2018;12:3–20. 29124875 10.1002/1878-0261.12155 PMC5748484

[29] Herber B, Truss M, Beato M, Muller R. Inducible regulatory elements in the human cyclin D1 promoter. Oncogene. 1994;9:1295–304. 8134134

[30] Tian H, Ge C, Li H, Zhao F, Hou H, Chen T, et al. Ribonucleotide reductase M2B inhibits cell migration and spreading by early growth response protein 1-mediated phosphatase and tensin homolog/Akt1 pathway in hepatocellular carcinoma. Hepatology. 2014;59:1459–70. 24214128 10.1002/hep.26929

[31] Tomas A, Futter CE, Eden ER. EGF receptor trafficking: consequences for signaling and cancer. Trends Cell Biol. 2014;24:26–34. 24295852 10.1016/j.tcb.2013.11.002 PMC3884125

[32] Berlin I, Sapmaz A, Stevenin V, Neefjes J. Ubiquitin and its relatives as wizards of the endolysosomal system. J Cell Sci. 2023;136(4):jcs260101. 10.1242/jcs.260101 PMC10022685 36825571

[33] Tang R, Langdon WY, Zhang J. Negative regulation of receptor tyrosine kinases by ubiquitination: Key roles of the Cbl family of E3 ubiquitin ligases. Front Endocrinol (Lausanne). 2022;13: 971162. 35966060 10.3389/fendo.2022.971162 PMC9365936

[34] Balak MN, Gong Y, Riely GJ, Somwar R, Li AR, Zakowski MF, et al. Novel D761Y and common secondary T790M mutations in epidermal growth factor receptor-mutant lung adenocarcinomas with acquired resistance to kinase inhibitors. Clin Cancer Res. 2006;12:6494–501. 17085664 10.1158/1078-0432.CCR-06-1570

[35] Yu HA, Arcila ME, Rekhtman N, Sima CS, Zakowski MF, Pao W, et al. Analysis of tumor specimens at the time of acquired resistance to EGFR-TKI therapy in 155 patients with EGFR-mutant lung cancers. Clin Cancer Res. 2013;19:2240–7. 23470965 10.1158/1078-0432.CCR-12-2246 PMC3630270

[36] Narayanan S, Cai CY, Assaraf YG, Guo HQ, Cui Q, Wei L, et al. Targeting the ubiquitin-proteasome pathway to overcome anti-cancer drug resistance. Drug Resist Updat. 2020;48: 100663. 31785545 10.1016/j.drup.2019.100663

