# Correction: PSMD9 promotes the malignant progression of hepatocellular carcinoma by interacting with c-Cbl to activate EGFR signaling and recycling

**DOI:** 10.1186/s13046-024-03209-2

**Published:** 2024-10-18

**Authors:** Yuting Su, Lili Meng, Chao Ge, Yuqi Liu, Chi Zhang, Yue Yang, Wei Tian, Hua Tian

**Affiliations:** 1 State Key Laboratory of Systems Medicine for Cancer, Shanghai Cancer Institute, Renji Hospital, Shanghai Jiao Tong University School of Medicine, 25/Ln 2200, Xietu Road, Shanghai, 200032 China; 2 Department of Pathology, Zhongshan Hospital, Fudan University, Shanghai, 200032 China; 3 Department of Pathology, The Afliated Hospital of Youjiang Medical University for Nationalities, Baise, 533000 China; 4 The Key Laboratory of Molecular Pathology (Hepatobiliary Diseases) of Guangxi, Baise, 533000 China

**Correction** **:** **J Exp Clin Cancer Res 43, 142 (2024)**

**https://doi.org/10.1186/s13046-024-03062-3**

Following publication of the original article [1], the authors identified errors Figure 1 and Figure 6. Incorrect images were obtained, specifically:

- Fig. 1M-1N - plot type errors were found on the hazard ratios of these figures. Figure 1M, HR value for Gender was mistakenly written as 0.184 instead of the correct value, which is 1.084.
- Fig. 6F - red fluorescence, which represents LAMP1, was erroneously labeled as EEA1

**Incorrect Fig.** 1

**Fig. 1:**
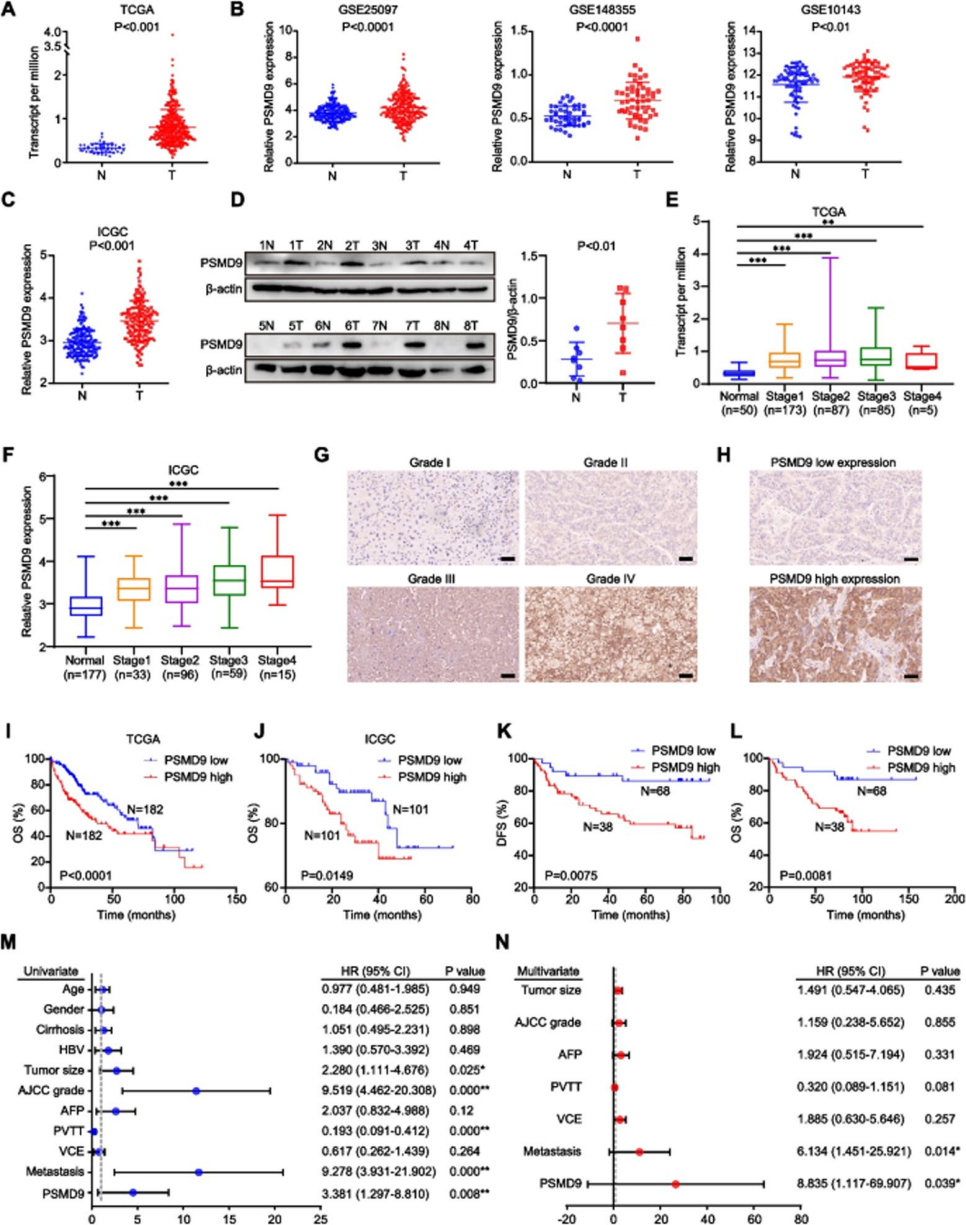
PSMD9 upregulation is associated with a poor prognosis in HCC patients. **A-C** The expression of PSMD9 in HCC tissues was compared with that in the corresponding noncancerous liver tissues in the TCGA datasets (*n* = 50) (**A**), the GSE10143, GSE25097 and GSE148355 (**B**) datasets and the ICGC-LIRI-JP dataset (**C**). **D** The expression of PSMD9 in HCC tissues was compared with that in the corresponding noncancerous liver tissues by Western blotting. **E–F** The expression of PSMD9 in noncancerous liver tissues and HCC tissues of diferent grades was analyzed in the TCGA (**E**) and ICGC-LIRI-JP cohorts (**F**). **G** Immunohistochemical analysis of PSMD9 expression in HCC samples. Representative images are shown. **H** Representative images of samples with high and low PSMD9 expression. **I-J** Overall survival analysis of HCC patients in the TCGA cohort (**I**) and the ICGC-LIRI-JP cohort (**J**) stratifed by the PSMD9 expression. **K-L** Overall and disease free survival analysis of 106 HCC patients stratifed by the PSMD9 expression level. **M–N** Univariate and multivariate Cox proportional hazards analyses were conducted to evaluate the HR of PSMD9 in terms of the overall survival of patients with HCC. *p < 0.05; **p < 0.01

**Correct Fig.** 1

**Fig. 1:**
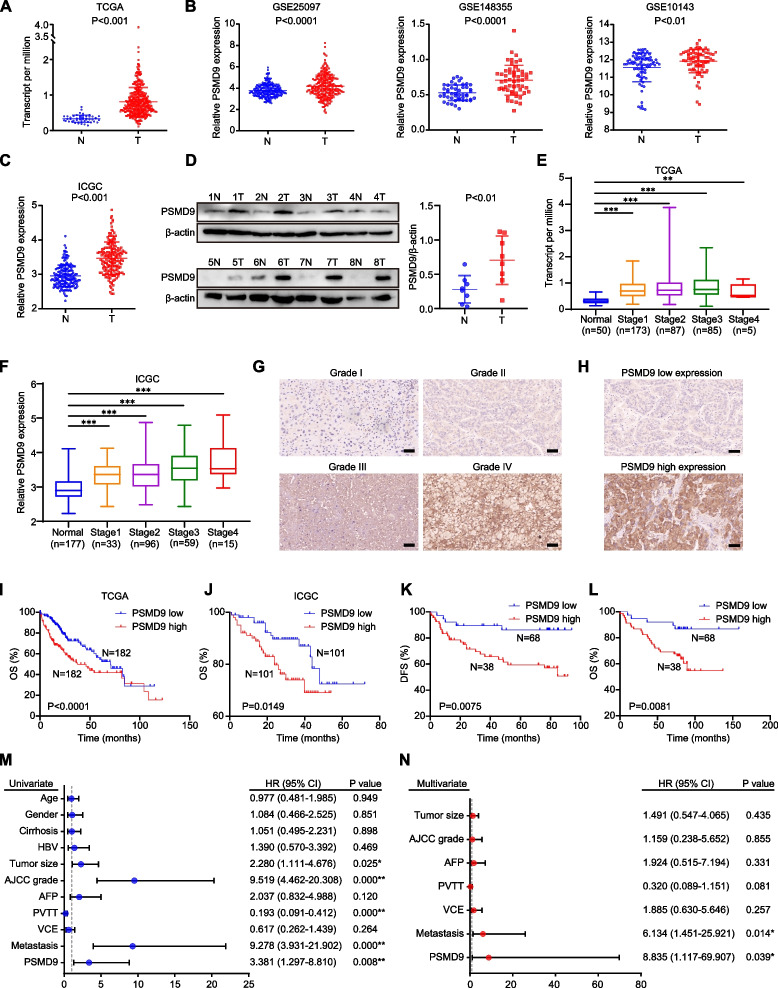
PSMD9 upregulation is associated with a poor prognosis in HCC patients. **A-C** The expression of PSMD9 in HCC tissues was compared with that in the corresponding noncancerous liver tissues in the TCGA datasets (*n* = 50) (**A**), the GSE10143, GSE25097 and GSE148355 (**B**) datasets and the ICGC-LIRI-JP dataset (**C**). **D** The expression of PSMD9 in HCC tissues was compared with that in the corresponding noncancerous liver tissues by Western blotting. **E–F** The expression of PSMD9 in noncancerous liver tissues and HCC tissues of diferent grades was analyzed in the TCGA (**E**) and ICGC-LIRI-JP cohorts (**F**). **G** Immunohistochemical analysis of PSMD9 expression in HCC samples. Representative images are shown. **H** Representative images of samples with high and low PSMD9 expression. **I-J** Overall survival analysis of HCC patients in the TCGA cohort (**I**) and the ICGC-LIRI-JP cohort (**J**) stratifed by the PSMD9 expression. **K-L** Overall and disease free survival analysis of 106 HCC patients stratifed by the PSMD9 expression level. **M–N** Univariate and multivariate Cox proportional hazards analyses were conducted to evaluate the HR of PSMD9 in terms of the overall survival of patients with HCC. *p < 0.05; **p < 0.01

**Incorrect Fig.** 6

**Fig. 6:**
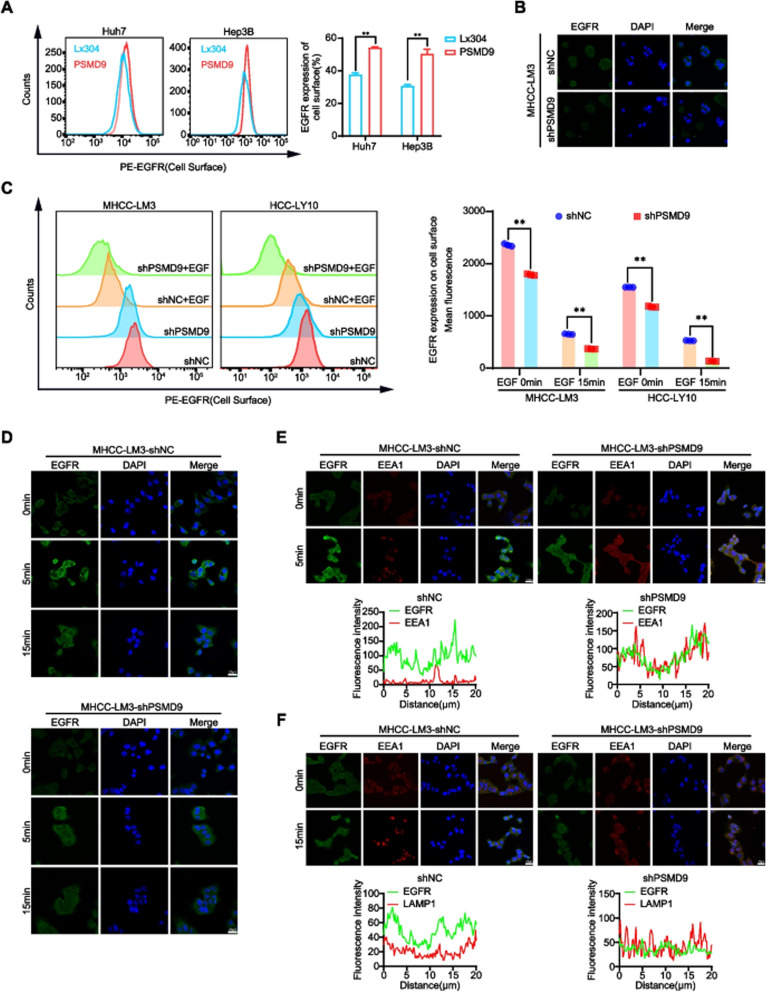
PSMD9 infuences EGFR endosomal trafcking. **A** EGFR expression on the cell surface was assessed by fow cytometry. **B** The expression of EGFR in PSMD9-knockdown MHCC-LM3 cells was assessed by immunofuorescence. **C** EGFR expression on the cell surface of PSMD9-knockdown MHCC-LM3 cells was assessed by fow cytometry in the presence of EGF. **D** The expression of EGFR in the presence of EGF for the indicated time periods was assessed by immunofuorescence. **E** PSMD9-knockdown MHCC-LM3 cells incubated with EGF for the indicated time periods were subjected to an immunofuorescence assay. Antibodies against EGFR and EEA1 were used. **F** PSMD9-knockdown MHCC-LM3 cells incubated with EGF for the indicated time periods were subjected to an immunofuorescence assay. Antibodies against EGFR and LAMP1 were used. **p < 0.01

**Correct Fig.** 6

**Fig. 6:**
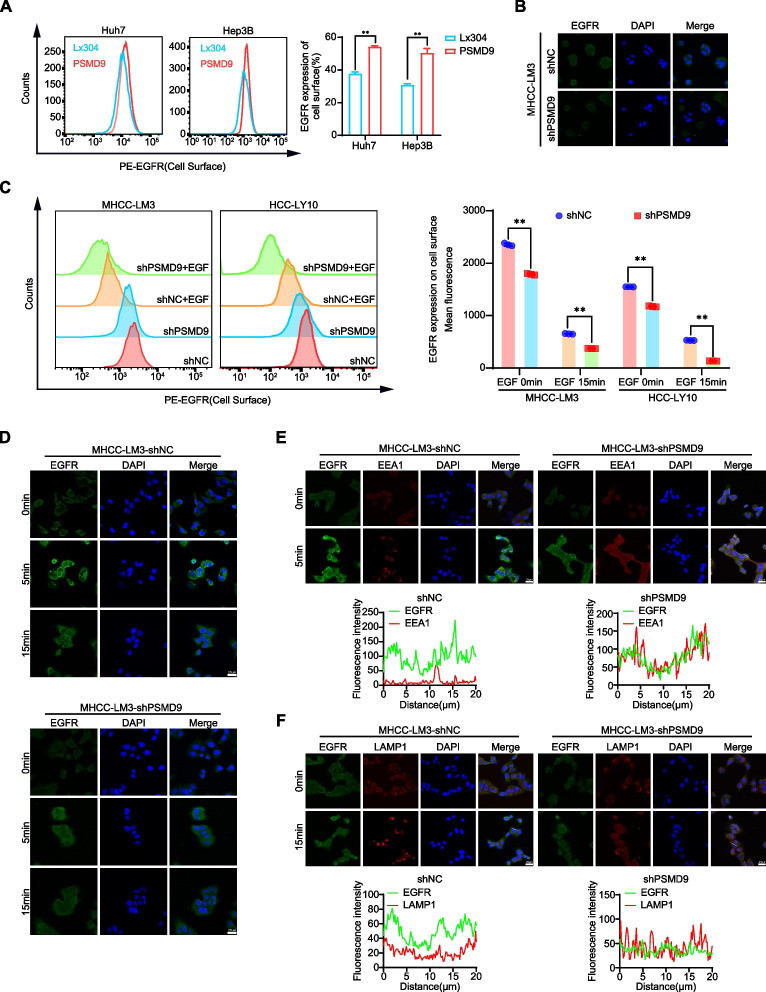
PSMD9 infuences EGFR endosomal trafcking. **A** EGFR expression on the cell surface was assessed by fow cytometry. **B** The expression of EGFR in PSMD9-knockdown MHCC-LM3 cells was assessed by immunofuorescence. **C** EGFR expression on the cell surface of PSMD9-knockdown MHCC-LM3 cells was assessed by fow cytometry in the presence of EGF. **D** The expression of EGFR in the presence of EGF for the indicated time periods was assessed by immunofuorescence. **E** PSMD9-knockdown MHCC-LM3 cells incubated with EGF for the indicated time periods were subjected to an immunofuorescence assay. Antibodies against EGFR and EEA1 were used. **F** PSMD9-knockdown MHCC-LM3 cells incubated with EGF for the indicated time periods were subjected to an immunofuorescence assay. Antibodies against EGFR and LAMP1 were used. **p < 0.01

The original article [1] has been corrected.
