# Supplementary material for: PSMD9 promotes the malignant progression of hepatocellular carcinoma by interacting with c-Cbl to activate EGFR signaling and recycling
Source: J Exp Clin Cancer Res. 2024 May 14;43:142. doi: 10.1186/s13046-024-03062-3 (PMC11092260; doi:10.1186/s13046-024-03062-3)
Supplement: Supplementary file 1 — Supplementary Material 1. [file 13046_2024_3062_MOESM1_ESM.docx]

#### Supplementary Table S1. The sequences of shRNA target

| Identifier | Forward(5’-3’) |
| --- | --- |
| PSMD9-1 | GCGCAGATCAAGGCCAACTAT |
| PSMD9-2 | GCGGGTCTGCAAGTGGATGAT |
| EGFR | CGCAAAGTGTGTAACGGAATA |

**Supplementary Table S2.**The sequences of gene-specific primers used for qRT-PCR.

| Gene name | Forward(5’-3’) | | Reverse (5’-3’) |
| --- | --- | --- | --- |
| **Primers for qRT-PCR** | | |  |
| PSMD9 | | ACCGCCAGGCACAACATC | TCATCCACTTGCAGACCCG |
| EGFR | | CGGGACATAGTCAGCAGTG | GCTGGGCACAGATGATTTTG |
| GAPDH | | AGAAGGCTGGGGCTCATTTG | AGGGGCCATCCACAGTCTT C |

**Supplementary Table S3.** Antibodies used in this study

| Antibody | catalog | | Dilution | | | Company |
| --- | --- | --- | --- | --- | --- | --- |
| **For Western blotting** | | | | | | |
| PSMD9 | 67338-1-Ig | 1:200 | | Proteintech | | |
| CDK4 | sc-56277 | 1:200 | | Santa Cruz | | |
| CDK6 | sc-7961 | 1:200 | | Santa Cruz | | |
| Cyclin D1 | sc-450 | 1:200 | | Santa Cruz | | |
| E2F1 | sc-251 | 1:200 | | Santa Cruz | | |
| Rb  p53  p-p53 ser15 | sc-102  sc-126  CST-9284 | 1:200  1:200  1:500 | | Santa Cruz  Santa Cruz  CST | | |
| p-Rb | CST-8516 | 1:1000 | | CST | | |
| PCNA | Ab29 | 1:1000 | | Abcam | | |
| EGFR | CST-4267 | 1:1000 | | CST | | |
| p-EGFR Tyr1068 | CST-3777 | 1:500 | | CST | | |
| ERK1/2 | Ab36991 | 1:1000 | | Abcam | | |
| p-ERK1/2 | CST-4073 | 1:1000 | | CST | | |
| Akt | CSt-4691 | 1:1000 | | CST | | |
| p-Akt | CST-4060 | 1:500 | | CST | | |
| Ub | sc-8017 | 1:200 | | Santa Cruz | | |
| c-Cbl | 25818-1-AP | 1:1000 | | Proteintech | | |
| STUB1 | CY8471 | 1:1000 | | Abways | | |
| Pakin | CY6641 | 1:200 | | Abways | | |
| β-actin | A3854 | 1:10000 | | Sigma | | |
| Secondary antibody | HRP conjugated goat anti-rabbit IgG | 1:4000 | | Sigma | | |
| Secondary antibody | HRP conjugated goat anti-mouse IgG | 1:4000 | | Sigma | | |
| **For Immunohistochemistry** | | | | | | |
| PSMD9 | 67338-1-Ig | 1:100 | | | Proteintech | |
| Mitochondria | ab92824 | 1:1000 | | | Abcam | |
| Ki67 | ab16667 | 1:500 | | | Abcam | |
| p-EGFR | CST-3777 | 1:100 | | | CST | |
| p-ERK1/2 | CST-4370 | 1:100 | | | CST | |
| Secondary antibody | Envision kit (HRP, rabbit/mouse, DAB+) | Ready-to-use | | | DAKO | |
| **For Immunofluorescence staining** | | | | | | |
| PSMD9 | 67338-1-Ig | 1:50 | | | Proteintech | |
| c-Cbl | 25818-1-AP | 1:50 | | | Proteintech | |
| EEA1 | 66218-1 | 1:50 | | | Proteintech | |
| LAMP1 | ab25630 | 1:50 | | | Abcam | |
| Secondary antibody | Alexa Fluor 594/488 anti-rabbit IgG | 1:50 | | | Invitrogen | |
| **For Flow cytometry** |  |  | | |  | |
| EGFR | 352903 | Ready-to-use | | | Biolegend | |

**Supplementary Table S4.**Clinicopathological features of HCC patients (106 cases)

| Clinicopathological features | Number |
| --- | --- |
| **Age** |  |
| ＜55 | 50 |
| ≥ 55 | 56 |
| **Gender** |  |
| Male | 82 |
| Female | 24 |
| **Tumor size** |  |
| ≤ 5 cm | 65 |
| >5 cm | 40 |
| **Cirrhosis** |  |
| Negative | 32 |
| Positive | 74 |
| **AFP (ng/mL)** |  |
| ≤20 | 33 |
| >20  Missing | 70  3 |
| **HBV** |  |
| Negative | 17 |
| Positive | 89 |
| **Portal vein tumor thrombus (PVTT)**  Negative  Positive  **Vessel carcinoma embolus (VCE)**  Negative  Positive  **Metastasis**  Negative  Positive  Missing | 93  13  33  73  88  17  1 |

**Supplementary Figure S1.** (A) The expression of PSMD9 in tumor tissues compared with paired corresponding noncancerous tissues was analyzed using data sets from TCGA. (B) The expression of PSMD9 in noncancerous liver tissues, tumor tissues and tumor tissues with metastasis was analyzed using data sets from TCGA. (C) Patients with high expression levels of PSMD9 had shorter DFS and OS than patients with low expression levels, as determined using data sets from TCGA and GSE54236.

**
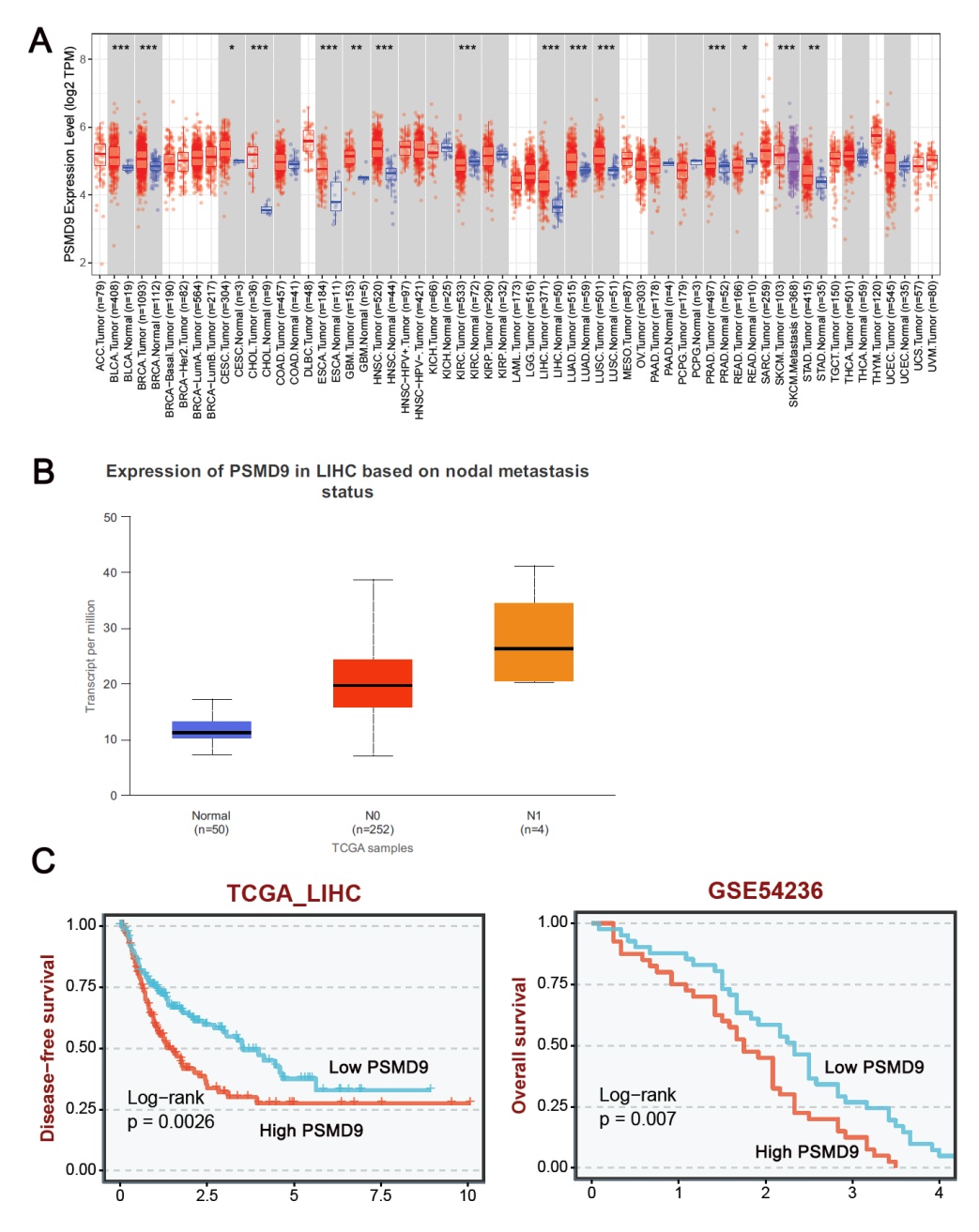
**

**Supplementary Figure S2.** Patients with high expression levels of PSMD9 had shorter overall survival than patients with low expression levels, as determined using data sets from TCGA.

**
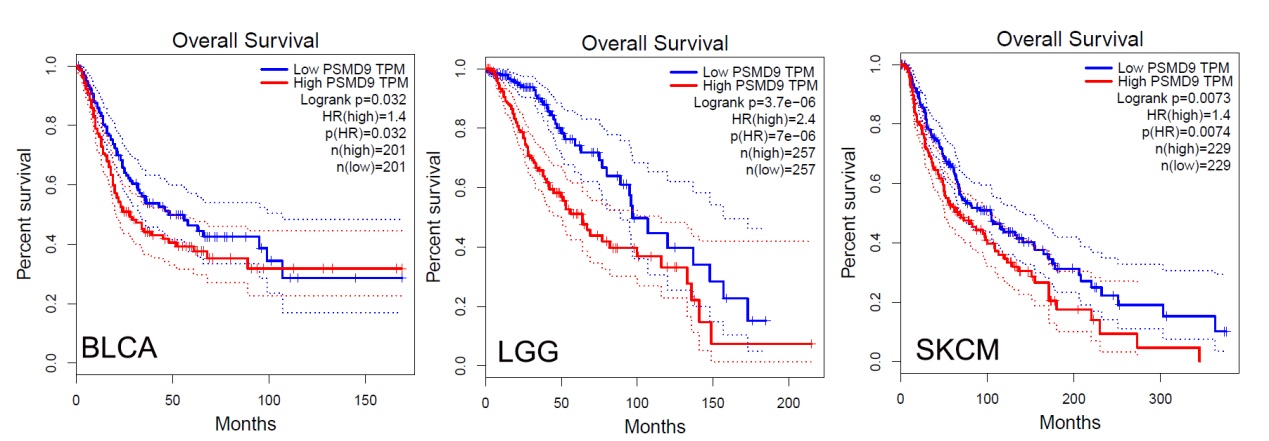
**

**Supplementary Figure S3.** (A)The expression of PSMD9 was analyzed by qPCR in HCC cell lines. (B) The expression of PSMD9 was analyzed by WB in HCC cell lines.

**Supplementary Figure S4.** (A) The expression of PSMD9 was detected by qPCR in PSMD9 knockdown HCC cells. (B) The effect of PSMD9 knockdown on HCC cell proliferation was assessed by EdU assay. (C) The expression of PSMD9 was detected by qPCR in PSMD9 overexpression HCC cells. (D) The effect of PSMD9 overexpression on HCC cell proliferation was assessed by EdU assay. (E) Expression of cell cycle related genes was detected in PSMD9 overexpression HCC cells by Western blotting.(F) Apoptosis was analyzed in PSMD9 overexpression HCC cells by flow cytometry.

**
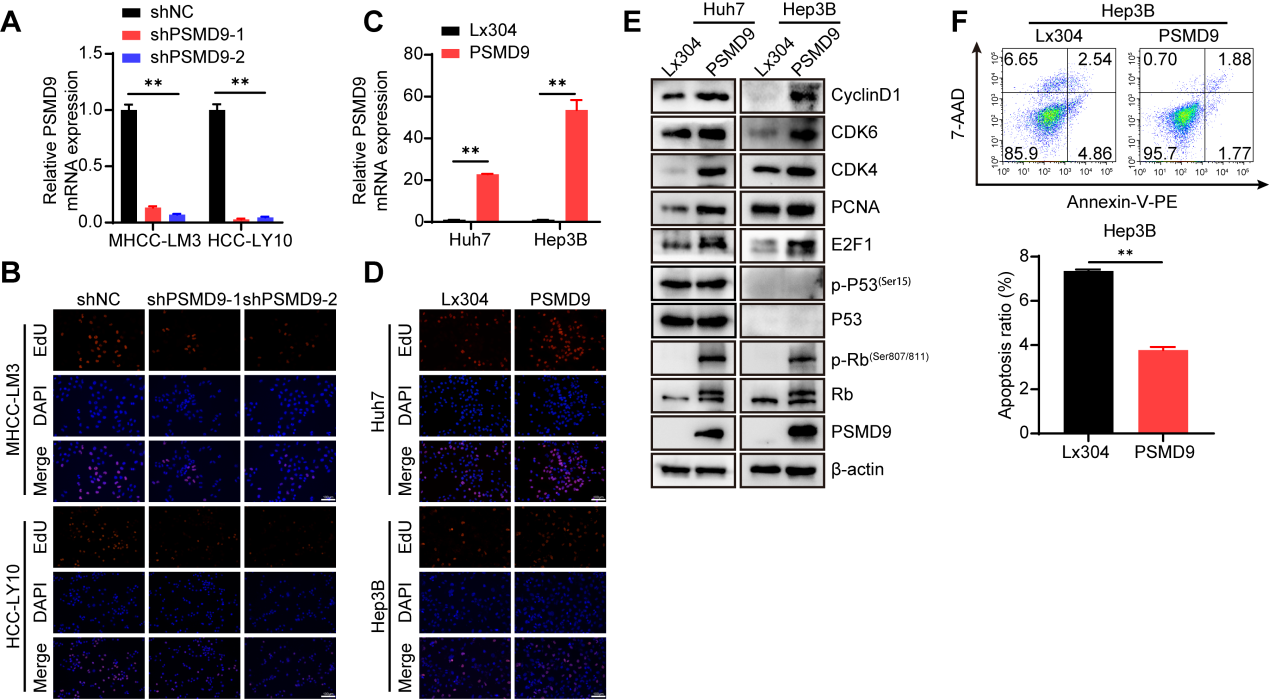
**

**Supplementary Figure S5.** (A) The expression of EGFR mRNA was analyzed by qPCR in PSMD9 overexpression and knockdown HCC cell lines. (B) CO-IP and Western blot showed that PSMD9 and c-Cbl bind to each other.

**Supplementary Figure S6.** (A-C) PSMD9 knockdown HCC cells were transfected with EGFR as indicated, and cell proliferation, migration and invasion were evaluated by EdU assay (A), colony formation (B), transwell asseys (C). (D-E) PSMD9-overexpressing HCC cells were treated with erlotinb or DMSO as indicated, and cell proliferation, migration and invasion were evaluated by EdU assay (D) and transwell assays (E)

**
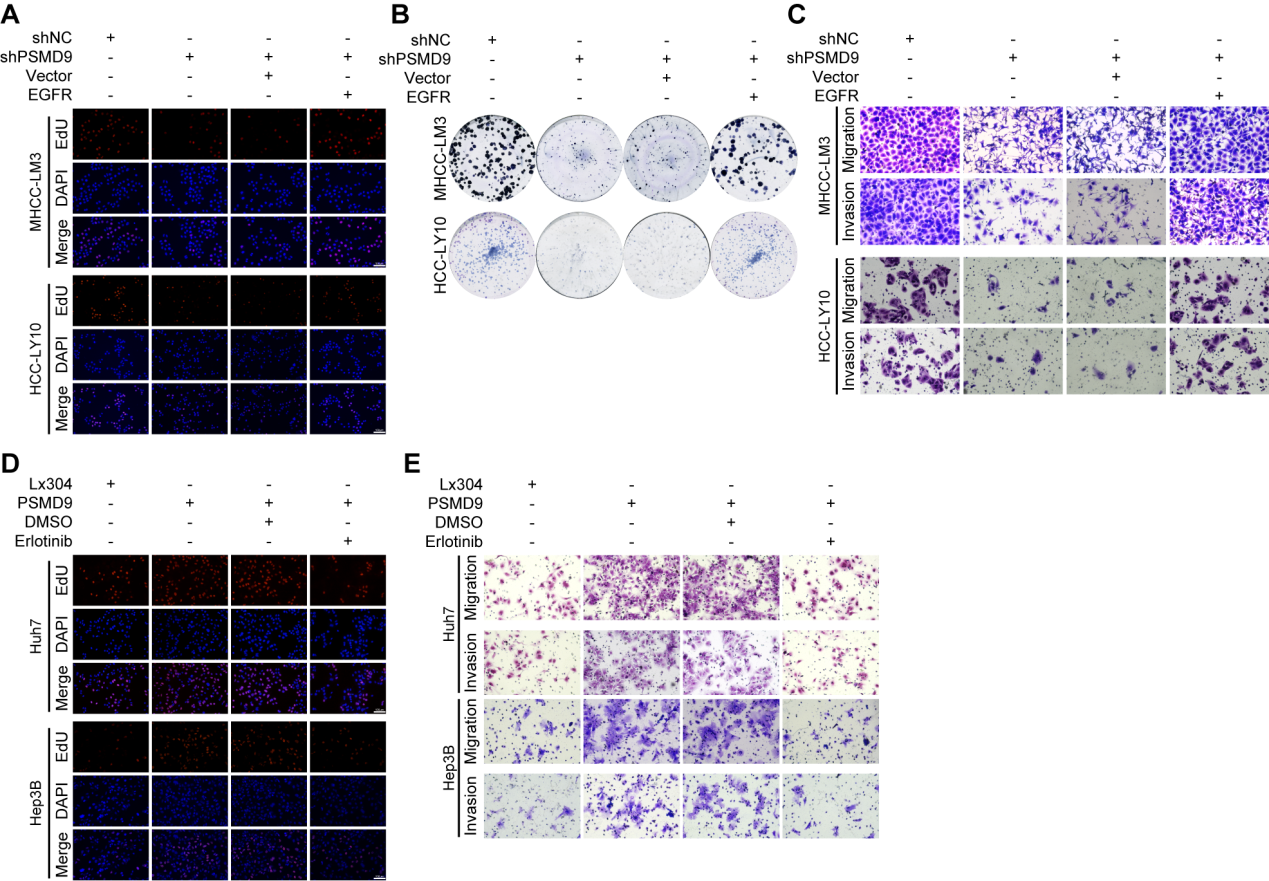
**

**Supplementary Figure S7.** (A) PSMD9-overexpressed HCC cells were transfected with EGFR shRNA, and the expression of EGFR and PSMD9 was detected by Western blotting. (B-E) PSMD9- overexpressed HCC cells were transfected with EGFR shRNA as indicated, cell proliferation, migration and invasion, and apoptosis were evaluated by EdU assay (B),CCK-8 assay (C), colony formation (D), transwell assays (E) and flow cytometry (F). **P<0.01.

**
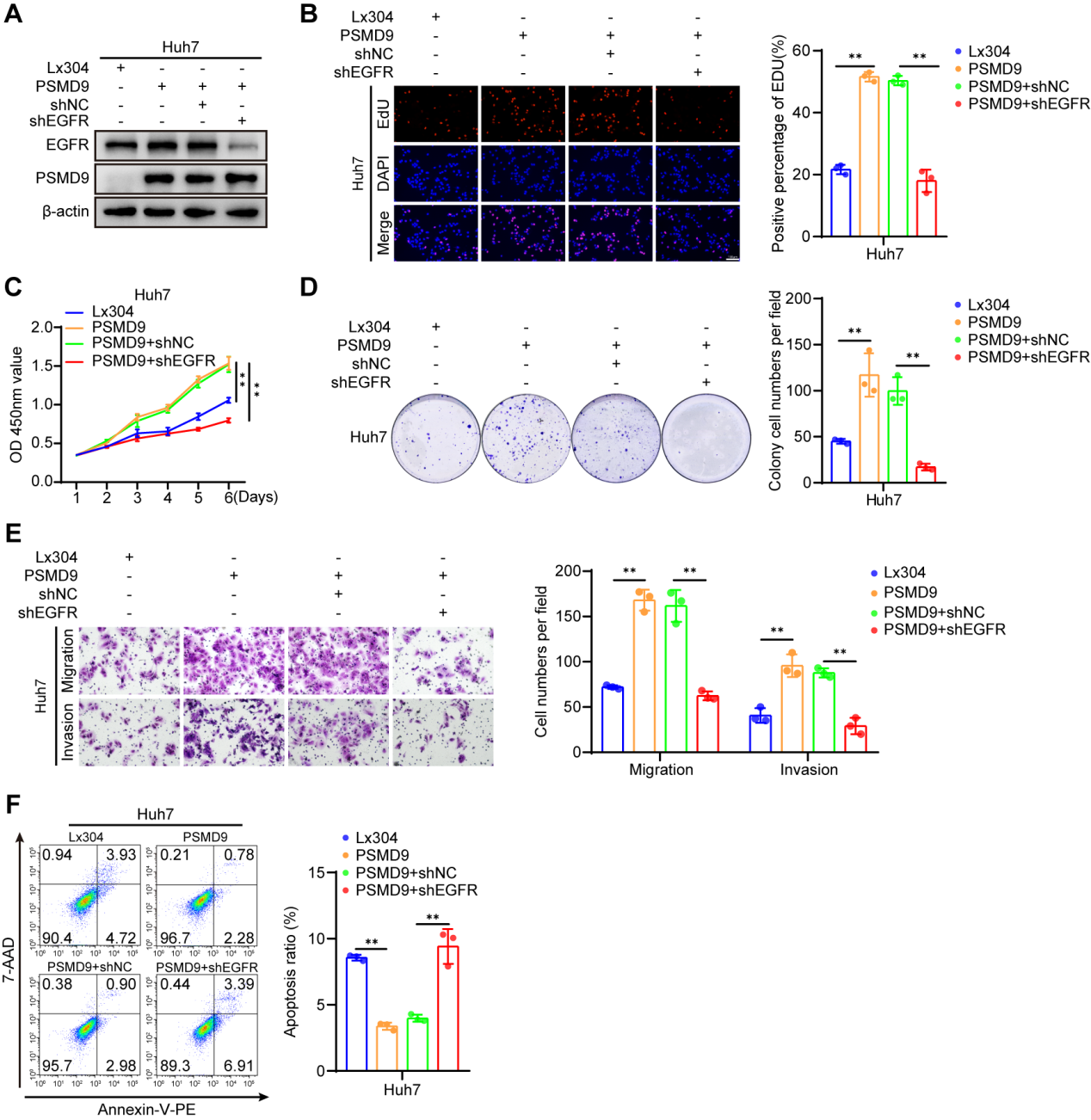
**

**Supplementary Figure S8.** (A-B) PSMD9 knockdown HCC cells were treated with erlotinb and cell proliferation were evaluated by EdU assay (A) and colony formation assays (B). (C) Migration and invasion was evaluated by transwell assay. (D) Body weight of mice after treatment with erlntinb or vehicle control.
